# Bacteria from the endosphere and rhizosphere of *Quercus* spp. use mainly cell wall-associated enzymes to decompose organic matter

**DOI:** 10.1371/journal.pone.0214422

**Published:** 2019-03-25

**Authors:** Ana V. Lasa, Tereza Mašínová, Petr Baldrian, Manuel Fernández-López

**Affiliations:** 1 Departament of Soil Microbiology and Symbiotic Systems, Estación Experimental del Zaidín, Consejo Superior de Investigaciones Científicas (CSIC), Granada, Spain; 2 Laboratory of Environmental Microbiology, Institute of Microbiology of the CAS, Praha, Czech Republic; Universite Paris-Sud, FRANCE

## Abstract

Due to the ability of soil bacteria to solubilize minerals, fix N_2_ and mobilize nutrients entrapped in the organic matter, their role in nutrient turnover and plant fitness is of high relevance in forest ecosystems. Although several authors have already studied the organic matter decomposing enzymes produced by soil and plant root-interacting bacteria, most of the works did not account for the activity of cell wall-attached enzymes. Therefore, the enzyme deployment strategy of three bacterial collections (genera *Luteibacter*, *Pseudomonas* and *Arthrobacter*) associated with *Quercus* spp. roots was investigated by exploring both cell-bound and freely-released hydrolytic enzymes. We also studied the potential of these bacterial collections to produce enzymes involved in the transformation of plant and fungal biomass. Remarkably, the cell-associated enzymes accounted for the vast majority of the total activity detected among *Luteibacter* strains, suggesting that they could have developed a strategy to maintain the decomposition products in their vicinity, and therefore to reduce the diffusional losses of the products. The spectrum of the enzymes synthesized and the titres of activity were diverse among the three bacterial genera. While cellulolytic and hemicellulolytic enzymes were rather common among *Luteibacter* and *Pseudomonas* strains and less detected in *Arthrobacter* collection, the activity of lipase was widespread among all the tested strains. Our results indicate that a large fraction of the extracellular enzymatic activity is due to cell wall-attached enzymes for some bacteria, and that *Quercus* spp. root bacteria could contribute at different levels to carbon (C), phosphorus (P) and nitrogen (N) cycles.

## Introduction

Globally, forests are considered relevant ecosystems since they provide essential environmental services; for instance, they contribute to the protection of biodiversity and soils [1]. Temperate deciduous forests contain approximately 0.66 trillion trees [2] and they represent a carbon (C) sink of high importance, especially in Europe, Asia and Northern America [3]. Therefore, the functioning of these ecosystems has been a research topic of great interest for long time. Forest microbiota–especially fungal and bacterial communities on plant litter, deadwood, soil and rhizosphere–contributes to the homeostasis of forest ecosystem by decomposing dead biomass, mediating the biogeochemical cycles and the nutrient turnover, and interacting with their plant hosts as symbionts or pathogens [4]. Although both saprotrophic and mycorrhizal fungal communities dwelling in forest soils have been studied exhaustively [5, 6], the knowledge of the involvement of bacteria in forest ecosystem processes remains fragmentary [4].

The degradation of lignocellulose is a vital step in the C cycle in terrestrial ecosystems since plant biomass is composed primarily of this complex mixture of biopolymers. Traditionally, fungi have been considered the main drivers of plant material decomposition [7], however there are progressively more works describing the potential contribution of bacteria to decomposition processes in forest litter and soils [8, 9]. Recently, Lladó et al. [10] described the ability of members of several bacterial phyla to degrade cellulose and hemicelluloses, which are the main non-polyphenolic constituents of lignocellulose. These authors revealed the important metabolic potential of Acidobacteria to degrade plant polysaccharides, while the cellulolytic ability of different bacterial genera isolated from a deciduous forest such as *Mucilaginibacter*, *Pedobacter* and *Luteibacter* was demonstrated in a genome- and proteome-based approach [11]. Several genera belonging to different orders of the phylum Actinobacteria (e.g. *Streptomyces*, *Cellulomonas*, *Acidothermus*, *Thermobifida*, *Cellulosimicrobium*, *Micrococcus*, *Clavibacter*, among others) [12–18] are also able to utilize complex biopolymers, namely cellulose or some types of hemicelluloses, although it has been suggested that this ability varies among strains [19]. Taken together, these results highlight the relevance of phylogenetically diverse bacteria in the global C cycle. Chitin is the second most abundant biopolymer on Earth just preceded by cellulose [20], as it is one of the main compounds of fungal cell walls and arthropod exoskeletons [21]. Many bacterial genera adscribed to different phyla are capable of producing chitinases to access the C and nitrogen (N) contained in this polymer, among which genera *Streptomyces* [22], *Glycomyces*, *Cellulomonas*, *Actinoplanes*, *Actinokineospora*, *Kitasatospora*, *Nonomurea* [23], *Pseudomonas*, *Ewingella*, *Pedobacter*, *Variovorax*, *Stenotrophomonas* and *Chitinophaga* are well known [24]. Thus, hydrolysis of chitin supposes a combination of an antagonism mechanism against soil fungi and a nutritional adaptation. Through the action of chitinases bacteria can degrade one of the main structural compounds of fungal cell wall during antagonistic interactions. However, the fact that bacteria are the most important decomposers of dead fungal mycelia [25] and N-acetalglucoasaminidase activity is increased during bacterial growth on this substrate [26] along with the fact that many bacteria feed on the final products of chitin decomposition, N-acetylglucosamine [10], indicate the nutritional role of chitin decomposition and the importance of chitinolytic bacteria in C and N cycles. Bacteria are also able to access one of the most limiting nutrients in many soils, phosphorus (P). As reviewed by Lladó et al. [4], members of several bacterial phyla dominated different processes related to P turnover, which include both the solubilization and uptake of inorganic P and the release of phosphate from organic sources through phosphatase enzymes. While these recent works shed some light on the activity of bacteria inhabiting litter and bulk soil, the information on bacterial inhabitants of the endospheres and rhizospheres of forest trees is scarce.

Organic matter transformation by bacteria is mediated by extracellular enzymes that are released into the soil or associated with the parent cell membrane, either attached to or integrated into it [27]. Once free-diffusible enzymes have been released into the surrounding environment, they face a set of hostile conditions [28]. Outside the cell, free enzymes may be subjected to adsorption, inactivation and degradation by proteases [29]. On the other hand, free enzymes may have higher probability to interact with their substrate [27]. While both cell-associated and free enzymes generate products available to all soil microorganisms, the producer cell has higher probability to access those produced close to its surface [30]. Furthermore, the fact that enzymes often contain substrate-binding domains [11] may allow bacteria with cell-bound enzymes to associate with their substrate, e.g. cellulose or other polysaccharides. As observed by Reintjes et al. [31] extracellular enzymes are produced preferentially as associated with cell membranes in marine bacteria, although the ecological relevance of the location of these enzymes has not been elucidated yet. In the rhizosphere–a highly competitive environment, especially in terms of nutrient uptake–those bacteria which have developed efficient nutritional strategies to compete against other microorganisms will likely become successful and proliferate. Thus, it could be expected that cell-bound enzymes will account for a large part of total activity in bacteria interacting with and having an effect on their plant hosts, such as rhizospheric and endospheric bacteria.

The aim of the present work was to characterize the ability of bacteria isolated from the rhizosphere and root endosphere of *Quercus* spp. trees to transform organic matter. Strains inhabiting the rhizosphere of burned *Quercus ilex* subsp. *ballota* and showing plant growth promotion traits were selected from a previous study [32], and those found in the root endosphere and rhizosphere of *Quercus pyrenaica* Willd. trees were also analysed. We tried to disentangle the contribution of both bacterial collections to forest ecosystem functionality by screening their ability to produce different enzymes involved in the C, N and P cycles. The spatial distribution of the extracellular enzymes with respect to producer cells was also assessed.

## Materials and methods

### Sample collection, bacterial isolation and identification

All bacterial strains characterized in this work were isolated from three different forests located in the Sierra Nevada National and Natural Park, in Southeast Spain. The Director of the Sierra Nevada National and Natural Park authorised the permission for the sampling. The field studies did not involve endangered or protected species. The geographic coordinates of each sampling location were N 36° 57’ 26”, W 3° 27’ 48” (BOF1); N 36° 57’ 11.2”, W 03° 26’ 21.0” (HAF1) and N 36° 58’ 23.4”, W 03° 24’ 36.4” (NPF1) (see S1 Table). The first site was covered by a holm oak (*Quercus ilex* subsp. *ballota*) forest located in the valley of the Lanjarón river, which was affected by a wildfire and where resprouting holm oak trees were found three years after the wildfire (BOF1, Burned Oak Forest). On the other hand, two sites located in the municipal district of Cáñar were selected: a natural, mature *Quercus pyrenaica* Willd. (melojo oak) forest located at the Higher Altitudinal limit of the Forest (HAF1) and another covered by a Scots pine (*Pinus sylvestris*) forest which was thinned out and naturalized with melojo oak plantlets in 2013 (NPF1, Naturalized Pine Forest). In each site, three trees with a diameter of at least 15 cm at breast height (in the case of BOF1 and HAF1 sites) and separated by more than 5 m were selected. BOF1 and HAF1 were the same sites described in a previous work [33, 34], and the positions of all the experimental areas were recorded with the Global Positioning System and are summarised in S1 Table.

Rhizospheric soil samples from holm oak (BOF1) and melojo oak trees (HAF1) were collected in previous works [33, 35]. Briefly, rhizospheric soil samples were taken by following the main roots until young cork-free roots were found, at a distance of approximately less than 50 cm from the trunk. To avoid the sampling of non-rhizospheric soil, the leaf litter from topsoil and minor roots from herbaceous plants were discarded collecting rhizospheric samples at depths from 5 to 25 cm. Samples coming from the three trees corresponding to the same site were mixed and sieved through a 2 mm mesh, and stored immediately at -80°C until processing them. Isolation and characterization of bacteria from holm oak rhizosphere was previously described by Fernández-González et al. [32]. Isolation of bacteria inhabiting the rhizosphere of melojo oak trees was performed with an initial pre-enrichment step where 5 g of soil obtained from melojo oak were resuspended in 50 mL mineral salts medium [36] supplemented with antipyrin, pyramidon, phenylalanine or tannic acid at a final concentration of 0.4%. After an incubation on a rotatory shaker for 7 days at 30°C, the biggest soil particles were removed. Afterwards, 100 μL of this soil suspension and the corresponding serial dilutions up to 10^−7^ were spread onto solid mineral salts medium supplemented with each of the aforementioned carbon sources. All the Petri dishes were incubated for 10 days at 30°C.

Endophytic bacteria were obtained from five melojo oak trees that were uprooted from NPF1 site two years after their planting. The roots were washed with water and ethanol and then put into clear plastic bags and conserved at 4°C until its processing. Roots from each tree were vigorously washed with tap water to remove the biggest soil particles attached to them and subsequently rinsed with distilled water. Afterwards, the young cork-free roots of each tree were cut into small pieces (roughly 5 cm of length) and surface sterilized for 1 min in ethanol 96%, 15 min in a solution containing 5% sodium hypochlorite, and 30 seconds in ethanol 96%. After rinsing the roots five times with sterile distilled water and cutting them into 5 mm pieces, 10 fragments were plated on YEM (Yeast Extract Mannitol) and TSA (Tryptic Soy Agar; Bacto, BD) solid media to verify the efficiency of the sterilization. On the other hand, 0.2 g of the remaining root material were soaked in liquid nitrogen and immediately ground for 6 min with the help of sterilized grinding balls (5 mm diameter) by using the grinder MM 031 (Retsch, Germany) at 30 Hz. The obtained root powder was mixed with 500 μL 20 mM phosphate buffer, and after a centrifugation step (2 min, 6 000 x g) 100 μL of each suspension was plated on YEM and TSA media in triplicate. All the plates–including those containing the sterilized roots–were incubated at 30°C for 7 days. The isolation of melojo oak bacterial endophytes was performed separately for each sampled tree.

The identification of those strains isolated from the rhizosphere of holm oak and melojo oak trees was performed previously by Fernández-González et al. [32] and Lasa et al., (unpublished), respectively, while endospheric strains were identified in this work. Briefly, in the case of the strains isolated from holm oak rhizosphere, the genomic DNA was obtained by suspending bacterial colonies in 100 μL sterile distilled water and heating them at 96°C for 10 min in a dry block heater. DNA of strains obtained from melojo oak trees (rhizo- and endosphere) was obtained after cultivating them in TSA and YEM liquid media, respectively, and by using the RealPure genomic DNA extraction kit (Durviz, S.L.U.,Valencia, Spain) and the corresponding manufacturer’s instructions. In all cases, isolated DNA was used as a template in PCR reactions to amplify the almost complete bacterial *rrs* gene by using the universal primers 9bfm [37] and 1512UR [38]. The specific PCR conditions were as follows: an initial denaturation step at 94°C for 4 min, 25 cycles of denaturation at 94°C for 1 min, primer annealing at 52°C for 1 min and extension at 72°C for 1.5 min, followed by a final step of heating at 72°C for 10 min. PCR products of the expected size were partially sequenced as described by Rivas *et al*., [39] on an ABI PRISM 3130xl Genetic Analyzer using a BigDye Terminator v3.1 Cycle Sequencing kit (Applied Biosystems Inc., USA) as recommended by the supplier. Obtained sequences were manually edited with the software Sequence Scanner v1.0 (Applied Biosystems, MA, EEUU) and compared against those sequences held in GenBank database through a BLASTn search https://blast.ncbi.nlm.nih.gov.

Among all the isolated strains, three bacterial collections were further analysed: the first, composed of 12 strains affiliated with genus *Arthrobacter* isolated from the rhizosphere of holm oak trees of which five may be keystone species in the recovery of burned holm oak forests, as demonstrated previously by their plant growth promotion ability [32]. 44 isolates belonging to genus *Pseudomonas* were also selected due to the relevance of family *Pseudomonadaceae* as one of the most potentially active taxa of *Q*. *pyrenaica*’s microbiota [35].The plant growth promoting traits and the ability to tolerate tannic acid *in vitro* of these strains led us to select them (Lasa *et al*., unpublished). On the other hand, among melojo oak root endophytes, 17 isolates of the genus *Luteibacter* were selected since it was the most abundantly isolated genus.

### Enzyme production of the selected bacterial strains

Since it is impossible to analyse enzyme production by individual bacterial strains directly in their environment (endosphere or rhizosphere), the 73 bacterial strains were cultivated on liquid Malt Extract medium (malt extract, 20 g L^-1^; 20 mL in 100-mL Erlenmeyer flasks). Malt extract was selected since it contains nutrients of plant origin that are relevant in the rhizosphere and endosphere of plant roots and in the soil including cellulose- and hemicellulose-derived oligosaccharides [40, 41]. Due to this, it was demonstrated to efficiently induce production of cellulases and hemicellulases in a wide range of bacteria, including Proteobacteria, Bacteroidetes, Actinobacteria but also in fungi [11, 19, 42–44]. Media containing long-chain polysaccharides had to be avoided since these are impossible to separate from bacterial cells and thus not suitable for detection and quantification of the cell wall-associated enzymes [44].

For each bacterial strain, triplicate flasks were inoculated with 200 μL of the corresponding inoculum previously adjusted to an optical density at a wavelength of 600 nm of 0.1, and incubated at 25°C on a rotatory shaker (200 rpm). After seven days, 10 mL of each bacterial culture were harvested and mixed, and the composite cultures were centrifuged for 3 minutes at 11 357 g. Moreover, after seven days all bacteria were at stationary phase of growth. Activity of cell-bound and free fractions of enzymes was determined as described previously [44]. Bacterial cells were washed twice with sodium acetate buffer (50 mM, pH 5.0) and resuspended in 20 mL of the same buffer. The culture supernatant was retained as well and its pH was adjusted by adding 10 mL of 50 mM acetate buffer, pH 5.0. Both cell fraction and culture supernatants were used to measure the activity of cell-attached and free enzymes, respectively.

The enzymatic activity of several enzymes that are relevant for nutrient cycling in forest ecosystems were studied in this work. Concretely, we assayed a collection of enzymes involved in the transformation of organic matter. The measurement of enzymatic activity was performed by using methylumberiferoll (MUF)-based fluorescent substrates as described by Baldrian [45] for the following enzymes: cellobiohydrolase (exocellulase, EC 3.2.1.91), β-glucosidase (EC 3.2.1.21), α-glucosidase (EC 3.2.1.20), β-xylosidase (EC 3.2.1.37), chitinase (EC 3.2.1.14), phosphomonoesterase (acid phosphatase, EC 3.1.3.2). The activities of α-galactosidase (EC 3.2.1.22), β-galactosidase (EC 3.2.1.23), β-glucuronidase (EC 3.2.1.31), β-mannosidase (EC 3.2.1.25), α-arabinosidase (EC 3.2.1.55) and lipase (EC 3.1.1.3) were assessed using 4-methylumbeliyferyl-α-D-galactopyranoside, 4-methylumbeliyferyl-β-D-galactopyranoside, 4-methylumbeliyferyl-β-D-glucuronide, 4-methylumbeliyferyl-β-D-mannopyranoside, 4-methylumbeliyferyl-α-L-arabinopyranoside and 4-methylumbeliyferyl-caprylate substrates, respectively, and the same procedure. In brief, in all cases MUF-labelled substrates dissolved in DMSO at a final concentration of 500 μM were mixed with samples (200 μL), in triplicate, in multiwell plates. Plates were incubated at 40°C and the fluorescence was measured from 5 to 125 minutes on a microplate reader (Infinite, TECAN, Austria), by using an excitation wavelength of 355 nm and an emission wavelength of 460 nm. Three technical replicates were measured, and the quantification of the activities was based on standard curves of 4-methylumbelliferone added to the samples after blank subtraction.

Cell-bound and supernatant fractions were analysed separately and the enzymatic activities were expressed per mL of bacterial culture. Total enzymatic activities were calculated as the sum of the activity in both fractions. The detection limit was 10 nM min^-1^ mL^-1^ and lower values were thus considered as a lack of activity.

### Statistical analyses

All statistical analyses were performed using R software 3.4.3 version [46]. To check if the enzymatic activity of each bacterial genus follows a normal distribution, the Shapiro test was used by means of the function *shapiro*.*test* included in R. As the normality of the data was not met, the non-parametric Mann-Whitney U test was applied to test if there were significant differences among the activity of cell-bound and released enzymes for each bacterial genus, considering all the strains that belonged to the same genus (*wilcox*.*test* function of R). Confidence levels > 95% (α = 0.05) were taken into account for all the statistical tests.

## Results

### Comparison of the activity of cell-associated and released enzymes

73 bacterial strains isolated from the root endosphere of melojo oak trees as well as from the rhizosphere of the same host and holm oak trees were studied in this work. Based on the partial sequence of the 16S rRNA gene, these strains showed the highest similarity percentage with genera *Luteibacter*, *Pseudomonas* and *Arthrobacter*, respectively (S2 Table).

**Fig 1:**
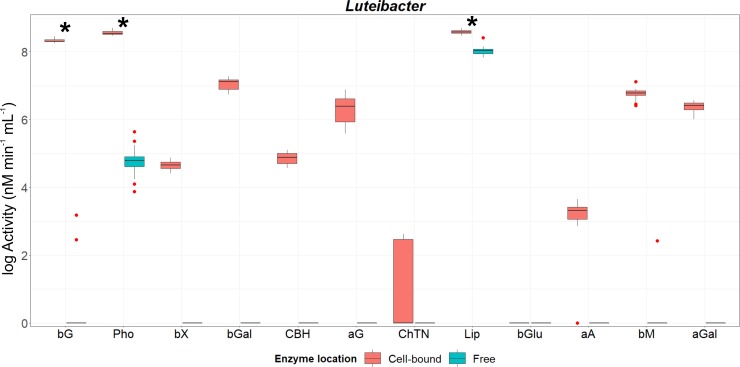
Activity of cell-associated and freely-released enzymes from genus *Luteibacter*. Box and whiskers were represented in the style of Tukey. Hinges correspond to the 1^st^ and 3^th^ quartiles and medians of the activity of each enzyme recorded for all strains are represented by horizontal lines. Red points correspond to those data beyond 1.5 x Inter Quartile Range. An ‘*’ indicates significant differences between the activity of those enzymes detected in both fractions calculated with the non-parametric Mann-Whitney U test at a confidence level of 95%. Abbreviations of enzymes: **bG:** β-glucosidase; **Pho:** acid phosphatase; **bX**: β-xylosidase; **bGal**: β-galactosidase; **CBH:** cellobiohydrolase; **aG**: α-glucosidase; **ChTN:** chitinase; **Lip:** lipase; **bGlu**: β-glucuronidase; **aA**: α-arabinosidase; **bM**: β-mannosidase; **aGal**: α-galactosidase. All the data were log-transformed for graphical clarity by fitting the same scale.

**Fig 2:**
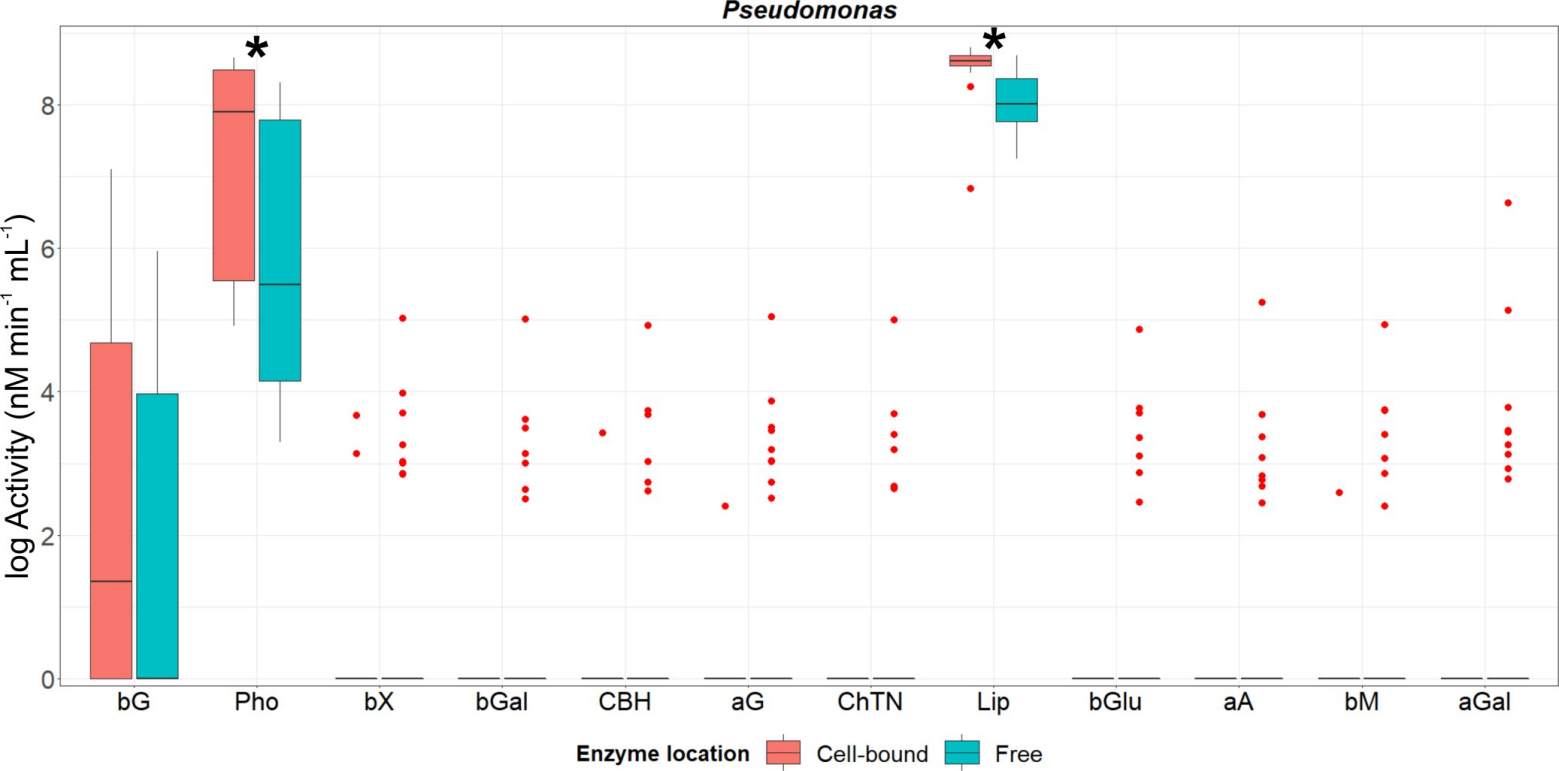
Activity of cell-associated and freely-released enzymes from genus *Pseudomonas*. Box and whiskers were represented in the style of Tukey. Hinges correspond to the 1^st^ and 3^th^ quartiles and medians of the activity of each enzyme recorded for all strains are represented by horizontal lines. Red points correspond to those data beyond 1.5 x Inter Quartile Range. An ‘*’ indicates significant differences between the activity of those enzymes detected in both fractions calculated with the non-parametric Mann-Whitney U test at a confidence level of 95%. Abbreviations of enzymes: **bG:** β-glucosidase; **Pho:** acid phosphatase; **bX**: β-xylosidase; **bGal**: β-galactosidase; **CBH:** cellobiohydrolase; **aG**: α-glucosidase; **ChTN:** chitinase; **Lip:** lipase; **bGlu**: β-glucuronidase; **aA**: α-arabinosidase; **bM**: β-mannosidase; **aGal**: α-galactosidase. All the data were log-transformed for graphical clarity by fitting the same scale.

**Fig 3:**
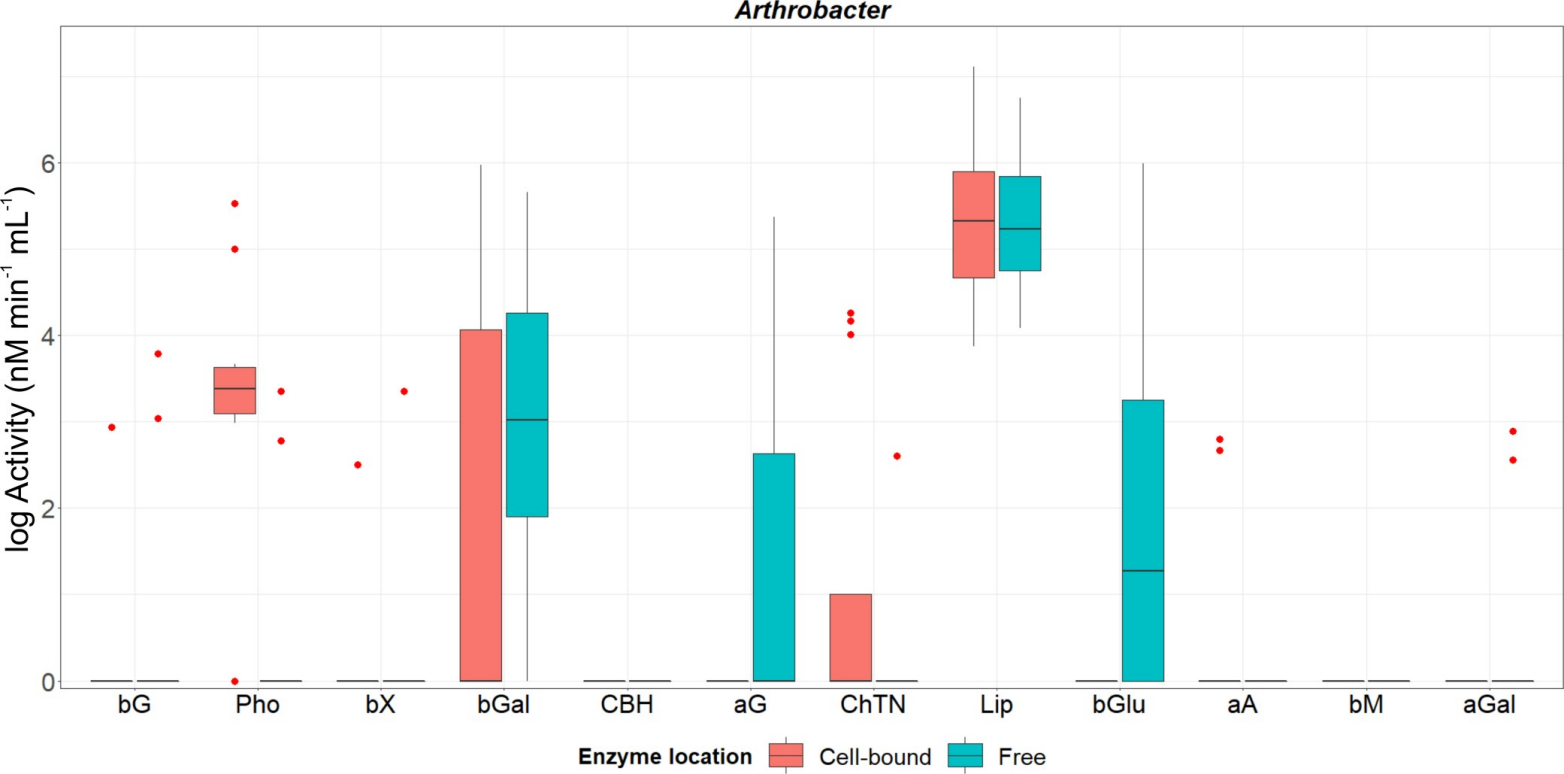
Activity of cell-associated and freely-released enzymes from genus *Arthrobacter*. Box and whiskers were represented in the style of Tukey. Hinges correspond to the 1^st^ and 3^th^ quartiles and medians of the activity of each enzyme recorded for all strains are represented by horizontal lines. Red points correspond to those data beyond 1.5 x Inter Quartile Range. No significant differences were detected between both fractions according to the non-parametric Mann-Whitney U test at a confidence level of 95%. Abbreviations of enzymes: **bG:** β-glucosidase; **Pho:** acid phosphatase; **bX**: β-xylosidase; **bGal**: β-galactosidase; **CBH:** cellobiohydrolase; **aG**: α-glucosidase; **ChTN:** chitinase; **Lip:** lipase; **bGlu**: β-glucuronidase; **aA**: α-arabinosidase; **bM**: β-mannosidase; **aGal**: α-galactosidase. All the data were log-transformed for graphical clarity by fitting the same scale.

The production of enzymes involved in the degradation of cellulose, hemicelluloses and other polysaccharides, as well as in the degradation of compounds based on N and P (chitin and phosphate esters, respectively) was assayed in both cell-wall associated and supernatant fractions (Figs 1–3 and S3 Table). For genus *Luteibacter*, all studied enzymes were detected in at least one fraction, except for β-glucuronidase, which was not produced in detectable amount in any of the studied fractions (Fig 1 and S4 Table). Overall, the enzyme activity was recorded mostly in the cell-bound fraction: seven enzymes were detected exclusively as attached to cells, and more than 97.6% of total activity of acid phosphatase, β-mannosidase and β-glucosidase recorded for all *Luteibacter* strains was associated with cells (S4 Table). Acid phosphatase was released into culture liquid by all isolates, however, those strains exhibiting highest activity in the culture liquid still had more than 95% of activity associated with its cells (see S4 Table). It was noticeable that for those enzymes that were synthesized as both cell-bound and free for at least two strains (β-glucosidase, acid phosphatase and lipase), the activity recorded in the cell-attached fraction was significantly higher than that detected in culture liquid (Mann-Whitney U test, p-values < 0.028; S3 Table).

The enzyme deployment strategy of *Pseudomonas* strains was not as definite as that of *Luteibacter* strains. On the one hand, five out of 12 enzymes were exclusively detected in the supernatant of *Pseudomonas* cultures (namely α-arabinosidase, β-galactosidase, chitinase, α-galactosidase and β-glucuronidase, Fig 2 and S3 Table). β-Mannosidase, exocellulase, β-xylosidase and α-glucosidase were synthesized in both fractions by just few strains, while the activity detected in the cell-associated fraction was considerable low (at most, 57.4% of the total activity of β-xylosidase in cell-bound fraction of strain p24; see S5 Table). On the other hand, significant differences between the activity of cell-associated and free enzymes were observed only for acid phosphatase and lipase, being the titres of both enzymes higher in the cell-bound fraction (S3 Table).

β-Mannosidase and exocellulase were not recorded at detectable levels in any of the *Arthrobacter* isolates tested. Although α-glucosidase, α-galactosidase and β-glucuronidase were produced exclusively as free-diffusible enzymes, less than 6.5% of the total activity of phosphatase and chitinase recorded for this genus was found in the culture liquid (S6 Table). α-Arabinosidase was the only enzyme which was found specifically in association with cells (S6 Table).

### Enzyme production potential of bacterial isolates

**Fig 4:**
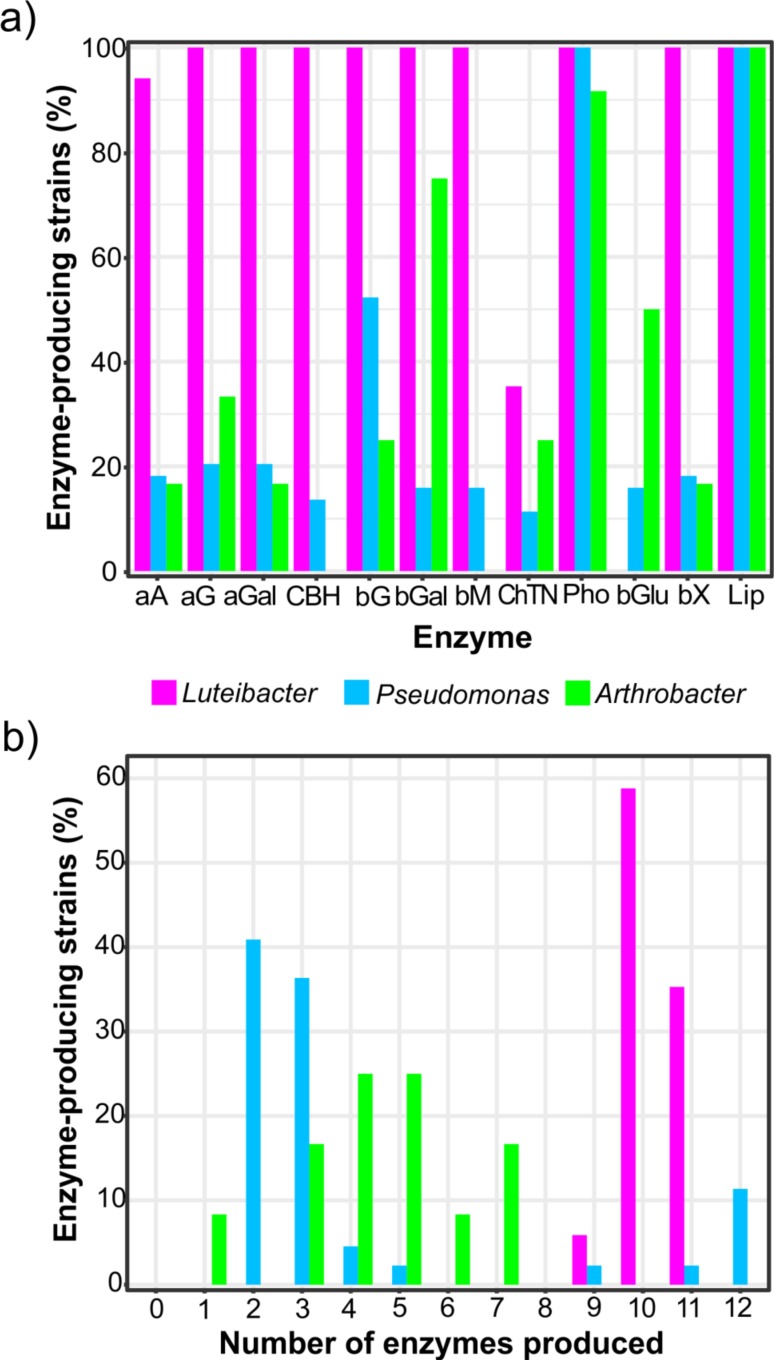
Relationship of the number of enzymatic activities and bacterial strains. (a) Percentage of *Luteibacter*, *Pseudomonas* and *Arthrobacter* strains exhibiting different enzymatic activities. (b) Enzymatic specialization of *Luteibacter*, *Pseudomonas* and *Arthrobacter* strains. The degree of specialization of each genus was measured as the total amount of strains producing different number of enzymes in liquid cultures. Abbreviations of enzymes: **aA**: α-arabinosidase; **aG**: α-glucosidase; **aGal**: α-galactosidase; **CBH:** cellobiohydrolase; **bG:** β-glucosidase; **bGal**: β-galactosidase; **bM**: β-mannosidase; **ChTN:** chitinase; **Pho:** acid phosphatase; **bGlu**: β-glucuronidase; **bX**: β-xylosidase; **Lip:** lipase.

**Fig 5:**
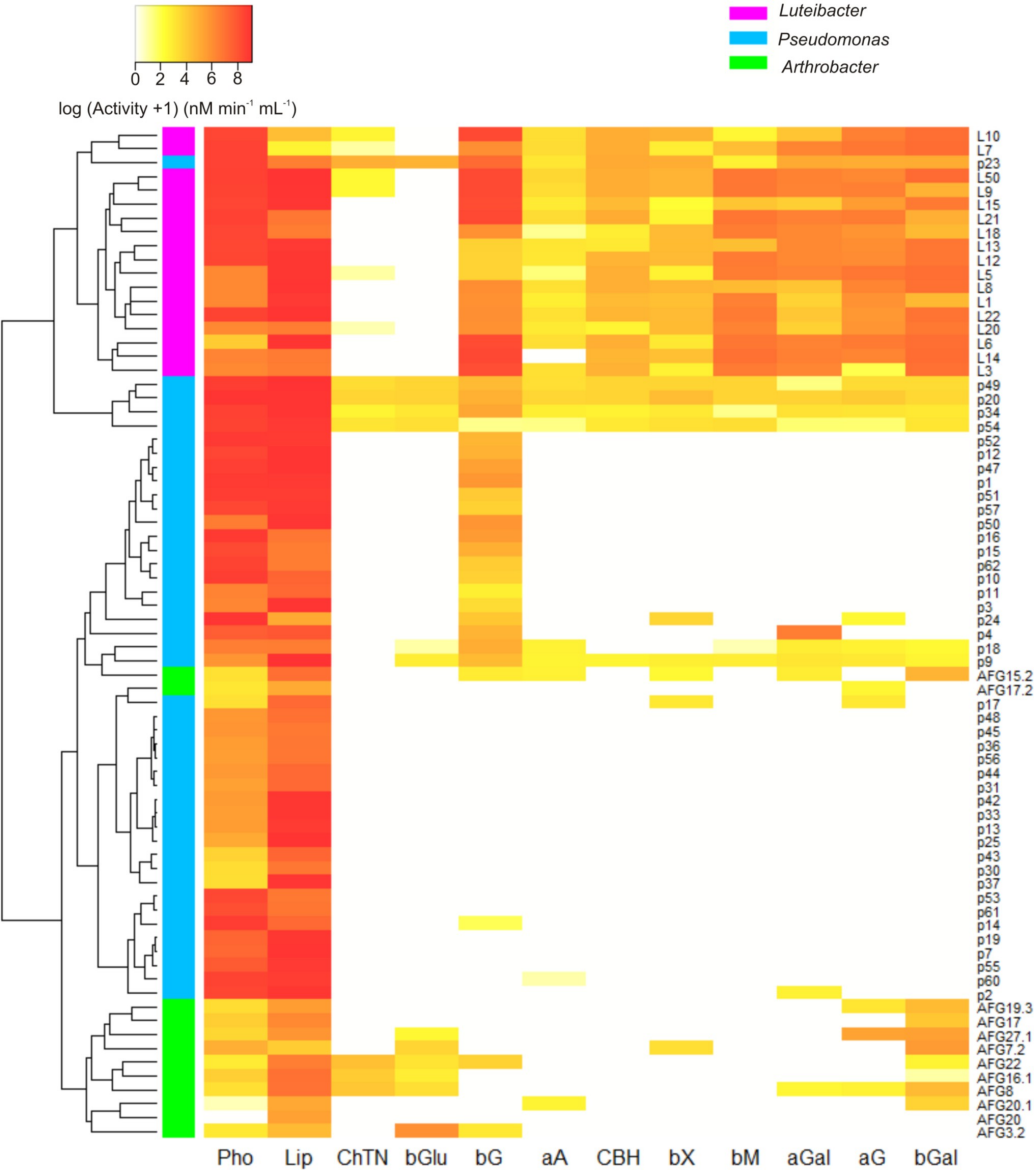
Enzymatic profiles of *Luteibacter Pseudomonas* and *Arthrobacter* strains. Data represent means of the total activity of three replicates and were log-transformed to fit the same scale. The clustering was constructed by using UPGMA algorithm based on Euclidean distances. Bacterial genera are colour-coded. Abbreviations of enzymes: **Pho:** acid phosphatase; **Lip**: lipase; **ChTN**: chitinase; **bGlu**: β-glucuronidase; **bG**: β-glucosidase; **aA**: α-arabinosidase; **CBH**: cellobiohydrolase; **bX**: β-xylosidase; **bM**: β-mannosidase; **aGal**: α-galactosidase; **aG**: α-glucosidase; **bGal**: β-galactosidase.

None of the *Luteibacter* isolates was able to produce β-glucuronidase and only 6 isolates were able to produce chitinase, however all the remaining enzymes were expressed by more than 94% isolates (Fig 4A). The enzyme production ability of those strains isolated from the root endosphere of *Q*. *pyrenaica* was noteworthy. Even for the least active strain L14 75% of assayed enzymes were detected. As shown in Fig 4B, 16 out of 17 isolates synthesized at least 10 enzymes, which resulted in an expression of roughly 86% of the tested enzymes on average. By contrast, the mean production of the isolates belonging to genus *Pseudomonas* did not exceed 33% of the enzymes assayed. Only a few strains contributed to a wide variety of enzymes, thus, just eight out 44 isolates were able to produce more than one third of the enzymes tested here (Fig 4B). Indeed, only five isolates (p20, p23, p34, p49, p54) were able to synthesize all the assayed enzymes, exhibiting the wider and more diverse range of enzymes produced (Fig 5). Nevertheless, these strains showed moderately low activity of all enzymes except for lipase, acid phosphatase, and β-glucosidase in some cases (S5 Table). On the other hand, any isolate belonging to *Arthrobacter* bacterial collection had the ability to produce all the enzymes, and those three isolates expressing more than a half of the assayed enzymes, produced seven enzymes at most (Fig 4B).

Although six *Luteibacter* strains were able to produce chitinase, its activity was close to the detection limit in all cases. In this sense, a low mean activity was also recorded for α-arabinosidase, yet it was commonly found among the isolates belonging to this genus (S4 Table). With the exception of the abovementioned enzyme, other enzymes involved in the decomposition of hemicellulose and also cellulose-degrading cellobiohydrolase were rather common and active in *Luteibacter* collection, specially β-galactosidase and β-mannosidase (S4 Table). Lipase, acid phosphatase and β-glucosidase exhibited the highest activities, as was observed for *Pseudomonas* collection. However, the latter was more rare among *Pseudomonas* isolates, being produced by approximately 50% of all of them. α-arabinosidase, exocellulase, β-galactosidase, β-mannosidase, chitinase, β-glucuronidase and β-xylosidase were expressed by less than 20% of *Pseudomonas* isolates (Fig 4A) and, besides, their activity was scarcely detected for most of the producing isolates. A lack of activity was reported for exocellulase and β-mannosidase among all the strains affiliated with genus *Arthrobacter*. Overall, typically low activity levels were detected for most of the enzymes and strains, even for acid phosphatase. Strikingly, the activity of this enzyme was on average around 88.5 and 64 times lower than that recorded for *Luteibacter* and *Pseudomonas* isolates, respectively. Again, lipase was the enzyme which on average exhibited the higher activity. It should be noted that this enzyme was the most widespread among the 73 strains studied; indeed, it was synthesized by all of them (Fig 4A).

## Discussion

Many attempts have been made in the past to gain more insights into the functionality of microorganisms in forest ecosystems, especially regarding fungal communities [47–49]. However, few efforts have been channelled towards nutrient cycles driven by cell-bound enzymes of soil bacteria. In this study, the role of 73 bacterial isolates in different ecosystem processes is reported by assessing the activity of cell-associated and free-diffusible enzymes involved in C, N and P cycles. The bulk of *Luteibacter*’s total activity (nearly 96.5% on average) was recorded in cell-bound fraction, and although the mean percentage of activity associated to cell-walls in *Pseudomonas* and *Arthrobacter* collections was lower (19.6 and 44.1%, S5 and S6 Tables, respectively), it was far from negligible. These results confirmed our main hypothesis, indicating the potential ability of the tested plant-associated bacteria to synthesize cell-associated enzymes. Although the production of cell-bound associated enzymes was mostly observed in the case of those strains isolated from melojo oak root endosphere (genus *Luteibacter*), we cannot conclude whether this pattern is explained by the endophytic origin of these strains or if it is a phenomenon associated to some specific genera. Differences in terms of extracellular enzymatic profiles have been previously observed for different species belonging even to the same genus, namely *Sphingomonas* and *Burkholderia* [10], so diverse enzyme deployment strategies could be expected among different bacterial genera. In order to address if the different location of extracellular enzymes is owing to the taxonomical diversity, further experiments including more number of endospheric and rhizospheric taxonomically diverse bacterial genera should be performed. In addition, since the resolution of the 16S rRNA gene sequence at intrageneric level is low [50]–especially for genus *Pseudomonas*–the correlation between enzyme deployment strategy and the taxonomy at species level is difficult to unravel. Thus, here we demonstrated that by ignoring the cell-wall associated enzymatic fraction, the total contribution of certain bacteria to organic matter decomposition could be underestimated.

Due to the location of the enzymes in the cell surface the degradation products are likely to remain in the vicinity of the producer cells, which could result in a reduction of the diffusional losses of the nutrients. By lessening the diffusion of hydrolysis products, the ‘cheating’ behaviour of those microorganisms which can intercept these products without producing extracellular enzymes could be mitigated or reduced [51]. As a result, the attachment of hydrolytic enzymes to cell walls could make its biosynthesis a more energetically cost-efficient process, and could represent an efficient nutritional strategy. Besides the nutritional implications of cell-bound enzymes for bacteria, activities associated with cell-walls result in a localized effect upon plant hosts. In the case of plant biomass degrading enzymes expressed by endophytes, the cell-bound location can restrain a generalized degradative process of host’s roots, thereby avoiding a boundless hydrolysis of plant tissues but allowing these bacteria a localized penetration into the host [52].

It is difficult to say how large is the fraction of cell-associated enzymes among other groups of bacteria or among microorganisms in general due to an almost total lack of data. The fact that high activity of free enzymes was detected in cultures of certain cellulolytic bacteria [11, 53] and soil Actinobacteria [19] indicates that the free fraction of enzymes can be relatively large. In the case of the highly active soil cellulose decomposer *Paenibacillus*, that efficiently degrades crystalline cellulose [53], it can be anticipated that its endocellulases attacking the crystalline domains of cellulose fibrils need to be freely mobile in order to access their substrate. In the screening of forest soil bacteria, Actinobacteria showed high enzyme production in culture while the values of Proteobacteria were low [10] which might indicate either low production, or association of enzymes with cells that was not tested in that case. For filamentous fungi that are able to cover decomposed substrates with their mycelia and thus ensure efficient capture of reaction products liberated by free enzymes, high activities of enzymes are typically found as free in their cultures [43]. There is, however, some evidence that some share of the total activity is still associated with mycelia [54]. In the case of yeasts, the majority of enzyme activity is associated with cells [44]. Due to their unicellularity, cell-association of enzymes may give them the same advantages as seen for bacteria. The question about the share of cell-bound activity in fungi, remains, however, undisclosed.

The activity of those enzymes involved in the breakdown of polysaccharides was almost exclusively detected as cell-associated in strains affiliated with genus *Luteibacter*; only three isolates expressed free β-mannosidase and β-glucosidase and the activity of these enzymes was residual in culture liquid. These observations were not unexpected since polysaccharases are often found as cell-associated enzymes [29]. Microbes have evolved several mechanisms to bind polysaccharides, such as carbohydrate binding modules included in cellulases and hemicellulases, and protruding structures like type IV pili. The latter mediate the adherence to cellulose in *Fibrobacter* and *Ruminococcus* species [55, 56], and it has been reported more recently in a cellulolytic *Luteibacter* strain isolated from the litter of a *Quercus petrea* forest [11]. Besides type IV pili, these authors also described the presence of glycoside hydrolases containing carbohydrate binding modules in the proteome of that *Luteibacter* strain. Taking into consideration our results and the adaptations to retain substrates in their proximity, we can speculate that the genus *Luteibacter* may have developed different strategies to improve nutrient acquisition, for instance by reducing the nutrient sharing with other bacteria. Moreover, the presence of decomposition products released by free enzymes could attract other bacteria, as occurred with root wounds [57]. Therefore, cell-associated enzymes could represent an adaptation developed by endophytic strains here studied belonging to genus *Luteibacter* to avoid such attraction of competitors to the root entry point. Notwithstanding the above, the importance of cell-wall attached enzymes of strains affiliated with this genus remains unclear and needs to be further explored to gain more insight about their physiology and ecology.

In addition to polysaccharide hydrolases, other hydrolases like lipase and acid phosphatase were synthesized as cell-attached in considerable proportions in all the *Luteibacter*, *Pseudomonas* and *Arthrobacter* collections. The production of these enzymes bound to cell-walls seems to be a widely distributed trait, since it has been previously observed in phylogenetically divergent microorganisms such as bacterial genera *Burkholderia* (class β-Proteobacteria) [58] and *Clostridium* (phylum Firmicutes) [59], and even in forest yeasts [44].

The location of the enzymes relative to parent cells could not always be certain but variable. *Pseudomonas* and *Arthrobacter* isolates did not show a production pattern as *Luteibacter* did. The activity of some enzymes was considerably high in cell-bound fraction for *Pseudomonas* and *Arthrobacter* isolates, representing more than 62 and 71% of the total activity, respectively (S5 and S6 Tables). However, freely-released enzymes ranged from 29.5 to 100% of the total activity in genus *Pseudomonas* whereas some enzymes were produced exclusively as free and other even not detected among *Arthrobacter* isolates. Although it has been already demonstrated that cell-associated enzymes accounted for the main part of total enzymatic activity in aquatic ecosystems, the contribution of free-diffusible enzymes is generally regarded as highly variable [27]. Many studies agree that the contribution of secreted enzymes relies on environmental factors even on different substrates [60, 61] and it should be pointed out that the location of hydrolytic enzymes varies across the time, life stage and from enzyme to enzyme [29] as observed here. Although free-enzymes suppose the diffusion and dilution of hydrolysis products from parent cells, other microbes could take advantage of this situation. The so-called ‘cheaters’ could grasp that soluble products, therefore, the expression of cell-free enzymes may exert a beneficial effect at community level. Thus, we suggest that some *Pseudomonas* and *Arthrobacter* isolates may have a syntrophic relationship with other microbial members in *Quercus* spp. rhizosphere, however we cannot define the specific role of those enzymes just with the results obtained in this work.

Traditionally, the enzyme location with respect to the producer cell has been overlooked when studying extracellular enzymes of soil microorganisms. Notwithstanding, it has allowed us to assess the contribution of bacteria that produced only cell-bound enzymes otherwise would not have been measured, especially in the case of those isolated affiliated with genus *Luteibacter*. By studying both locations and taking into account a historically almost ignored enzyme location for many soil bacteria (cell-bound attached fraction), the potential involvement of each genus in nutrient turnover was determined. Overall, *Pseudomonas* and *Arthrobacter* isolates showed a moderated range of enzymes produced and relatively low levels of enzymatic activity, which indicates that most of the isolates of these genera have the potential to contribute to a limited soil processes to a greater or lesser extent.

Effective cellulose degradation typically relies on exocellulase (cellobiohydrolase) and β-glucosidase, which yields glucose from cellobiose (a product of cellulose hydrolysis). *Pseudomonas* p23 and all *Luteibacter* isolates exhibited high activity not only of β-glucosidase, but also of exocellulose, which catalyzes the rate-limiting step of cellulose deconstruction, suggesting these strains are truly involved in the decomposition of this polymer. Berlemont and Martiny [62] reported in a genome-based approach that both genes encoding exocellulases and β-glucosidases were distributed in 24% of all bacterial genomes, and the majority of the lineages (56%) encoded only genes for β-glucosidases in terms of cellulose hydrolysis. Our results are partially in accordance with these observations: the expression of both exocellulase and β-glucosidase was moderately common among isolates (31.5%), however the proportion of strains exhibiting exclusively β-glucosidase activity only reached half of the predicted value, probably because gene content does not necessarily mean gene expression. Those isolates producing only β-glucosidase could be considered opportunistic bacteria since they could be taking advantage of the cellobiose generated by exocellulase producing microorganisms. Therefore, our results suggest that *Luteibacter* and several *Pseudomonas* isolates may be cellulolytic bacteria, either as truly decomposers or as opportunistics. Actinobacteria are generally considered as an important phylum in cellulose degradation in forest soils [4, 63], and although only few lineages are able to degrade this polymer, cellobiose utilization is rather common in this phylum. Indeed, only 20% of actinobacterial genomes do not contain genes encoding any enzyme involved in cellulose degradation [62]. In spite of the fact that the cellulolytic capability of some strains affiliated with genus *Arthrobacter* have already been described [64], our isolates did not produce exocellulase and only three isolates exhibited very low β-glucosidase activity. It is possible that many strains harbouring exocellulase and β-glucosidase genes do not express them or that *Arthrobacter* isolates studied here represent an exception in cellulose degradation.

Although the genus *Luteibacter* is not well characterized yet and some authors have previously reported that strains isolated from a terrestrial lichen are not even able to produce β-glucosidase [65], recently it has been described as cellulolytic [11]. These authors reported its ability to degrade also hemicellulose and detected different hemicellulose-degrading proteins in its proteome. As shown in S4 Table, *Luteibacter* isolates resulted active producers of hemicellulose degrading enzymes, specially β-mannosidase and β-galactosidase, thereby agreeing with the observations of López-Mondéjar et al. [11]. It is worth noting the diversified enzymatic competence exhibited by *Luteibacter* isolates which indicates these endophytes are not specialized decomposers and have evolved a diverse enzymatic system. This set of different but complementary enzymes could act synergistically and contribute altogether to a more efficient hydrolysis of plant cell wall compounds. Extracellular enzymes involved in plant cell wall degradation (e.g. cellulases, hemicellulases, xylanases, pectinases, polygalacturonases, amylases) are commonly synthesized by endophytic bacteria to penetrate in plant tissues [66, 67]. Other bacterial taxa isolated from root endosphere of cotton or even mangrove trees have the ability to produce a wide variety of enzymes which aid these endophytes in penetrating plant roots [57, 68]. Therefore, it would not be striking that the expression of cellulases and hemicellulases observed for *Luteibacter* was related to its endophytic origin; nevertheless additional confirmatory experiments should be performed to clarify the concrete role of these enzymes for those strains.

The production of acid phosphatase was common for all isolates studied here. According to Bergkemper and colleagues [69], phylogenetically diverse bacteria were commonly involved in P turnover in temperate forest soils, among which order Xhantomonadales (to which genus *Luteibacter* belongs) dominated the expression of acid phosphatase. Lladó et al. [10] also reported high activity levels of this enzyme for isolates belonging to different phyla in a coniferous forest, while several *Pseudomonas* species are well-known for their role in the release of phosphate in a wide variety of agricultural soils. *Arthrobacter nanjingensis* is another example of a bacterium isolated from forest environment which also showed acid phosphatase activity [70], thereby highlighting the theoretical important role of these taxa in P turnover.

## Conclusions

So far, enzymes bound to cells have rarely been studied in detail in soil bacteria. As reported here, the activity of enzymes attached to cells involved in organic matter decomposition can suppose a substantial part of their total extracellular enzymatic activity; indeed, for some strains they can be detected exclusively bound to cells. Our results suggested that measuring only freely diffusible enzymes, the total enzymatic potential of many root-associated bacteria could be underestimated. Hence, here we suggest taking heed of cell-bound fraction for further analyses so that the involvement of bacteria in nutrient cycling processes mediated by extracellular enzymes could be described in a more reliable way. By studying both fractions, we have been able to describe the potential of *Luteibacter* isolates as cellulolytic and hemicellulolytic bacteria, whereas the potential contribution of genera *Pseudomonas* and *Arthrobacter* to plant biomass decomposition processes was moderate and subtle, respectively. In short, our results reflected different ecological significance of both three genera in terms of C, N and P cycling, and pointed out the importance of measured cell-bound enzymes.

## Supporting information

S1 Table Position and main characteristics of the experimental areas. (PDF)

S2 Table Identification of the strains studied based on their partial 16S rRNA gene sequence. The identity of the closest hit for each strain is indicated, and in some cases, several hits are reported for those strains which showed the same similarity percentage with different species.
(PDF)

S3 Table Activity of cell-wall bound and freely-released enzymes produced by genera Luteibacter, Pseudomonas and Arthrobacter isolated from the root endosphere or the rhizosphere of Quercus spp. trees. Data represent means and standard deviations of activities of those enzymes produced by two or more strains. A ‘-’ indicates values below detection limit. An ‘*’ indicates values that were recorded just for one strain, the one which is shown in parentheses. Abbreviations of enzymes: **bG**: β-glucosidase; **Pho**: acid phosphatase; **Lip**: lipase; **bM**: β-mannosidase; **aA**: α-arabinosidase; **bX**: β-xylosidase; **bGal**: β-galactosidase; **CBH**: cellobiohydrolase; **aG**: α-glucosidase; **ChTN**: chitinase; **aGal**: α-galactosidase; **bGlu**: β-glucuronidase. For each bacterial genus, significant differences between the activity of those enzymes detected in both fractions were calculated by using the non-parametric Mann-Whitney U test at a confidence level of 95%.
(PDF)

S4 Table Activity of cell-bound and freely-released enzymes, and total enzymatic activity of strains of genus Luteibacter. Data represent means and standard deviations of the total activity of three replicates. Abbreviations of enzymes: **bG**: β-glucosidase; **Pho**: acid phosphatase; **Lip**: lipase; **bM**: β-mannosidase; **aA**: α-arabinosidase; **bX**: β-xylosidase; **bGal**: β-galactosidase; **CBH**: cellobiohydrolase; **aG**: α-glucosidase; **ChTN**: chitinase; **aGal**: α-galactosidase; **bGlu**: β-glucuronidase. A ‘-’ indicates values below detection limit
(PDF)

S5 Table Activity of cell-bound and freely-released enzymes, and total enzymatic activity of strains of genus Pseudomonas. Data represent means and standard deviations of the total activity of three replicates. Abbreviations of enzymes: **bG**: β-glucosidase; **Pho**: acid phosphatase; **Lip**: lipase; **bM**: β-mannosidase; **aA**: α-arabinosidase; **bX**: β-xylosidase; **bGal**: β-galactosidase; **CBH**: cellobiohydrolase; **aG**: α-glucosidase; **ChTN**: chitinase; **aGal**: α-galactosidase; **bGlu**: β-glucuronidase. A ‘-’ indicates values below detection limit.
(PDF)

S6 Table Activity of cell-bound and freely-released enzymes, and total enzymatic activity of strains of genus Arthrobacter. Data represent means and standard deviations of the total activity of three replicates. Abbreviations of enzymes: **bG:** β-glucosidase; **Pho**: acid phosphatase; **Lip**: lipase; **bM**: β-mannosidase; **aA**: α-arabinosidase; **bX**: β-xylosidase; **bGal**: β-galactosidase; **CBH**: cellobiohydrolase; **aG**: α-glucosidase; **ChTN**: chitinase; **aGal**: α-galactosidase; **bGlu**: β-glucuronidase. A ‘-’ indicates values below detection limit.
(PDF)
