# Supplementary material for: Bacteria from the endosphere and rhizosphere of Quercus spp. use mainly cell wall-associated enzymes to decompose organic matter
Source: PLoS One. 2019 Mar 25;14(3):e0214422. doi: 10.1371/journal.pone.0214422 (PMC6433265; doi:10.1371/journal.pone.0214422)
Supplement: S1 Table — (PDF) [file pone.0214422.s001.pdf]

S1 Table. Position and main characteristics of the experimental areas.

Site	Location	Altitude (masl)	Site description	Orientation	References
**HAF1**	N 36° 57' 11.2'' W 03° 26' 21.0''	1778	Natural, mature forest of *Quercus pyrenaica* Willd. (melojo oak) located at the **H**ighest **A**ltitudinal limit of the **F**orest	South facing	Cobo-Díaz *et al.*, 2015
**BOF1**	N 36° 57' 26'', W 3° 27' 48''	1566	**B**urned *Quercus ilex* subsp. ballota (evergreen holm **O**ak) **F**orest covered by re-growing oak trees after the wildfire	South facing	Cobo-Díaz *et al.*, 2015; Fernández-González *et al.*, 2017
**NPF1**	N 36º 58' 23.4'', W 03º 24' 36.4''	1887	**P**ine **f**orest which was thinned out and **n**aturalized by planting *Q. pyrenaica* plantlets	South facing	This work

masl, meters above the sea level
