# Supplementary material for: Bacteria from the endosphere and rhizosphere of Quercus spp. use mainly cell wall-associated enzymes to decompose organic matter
Source: PLoS One. 2019 Mar 25;14(3):e0214422. doi: 10.1371/journal.pone.0214422 (PMC6433265; doi:10.1371/journal.pone.0214422)
Supplement: S2 Table — The identity of the closest hit for each strain is indicated, and in some cases, several hits are reported for those strains which showed the same similarity percentage with different species. (PDF) [file pone.0214422.s002.pdf]

S2 Table. Identification of the strains studied based on their partial 16S rRNA gene sequence. The identity of the closest hit for each strain is indicated, and in some cases, several hits are reported for those strains which showed the same similarity percentage with different species.

Strain[a]	Accesion number (query)	Most similar classified strain	Accesion number [best hit(s)]	Similarity (%)
**L1**	MK559965	*Luteibacter rhizovicinus* LJ96^T	CP017480	100.00
**L3**	MK559965			
**L5**	MK559965			
**L6**	MK559965			
**L7**	MK559965			
**L8**	MK559965			
**L9**	MK559965			
**L10**	MK559965			
**L12**	MK559965			
**L13**	MK559965			
**L14**	MK559965			
**L15**	MK559965			
**L18**	MK559965			
**L22**	MK559965			
**L20**	MK559963	*Luteibacter rhizovicinus* LJ96^T	CP017480	99.80
**L21**	MK559964			
**L50**	MK559966	*Luteibacter rhizovicinus* LJ96^T	CP017480	99.87
**p1**	MK559906	*Pseudomonas koreensis* PgBE56	MH144278	98.92
		Pseudomonas fluorescens LBUM677	MG461479	98.92
		Pseudomonas moraviensis I5-6	KU229982	98.92
**p2**	MK559907	*Pseudomonas mandelii* YF11-3(1)	MH144323	99.93
**p3**	MK559908	*Pseudomonas koreensis* PgBE36	MH144259	99.00
		Pseudomonas fluorescens LBUM677	MG461479	99.00
**p4**	MK559909	*Pseudomonas koreensis* PgBE36	MH144259	99.00
		Pseudomonas fluorescens LBUM677	MG461479	99.00
**p7**	MK559910	*Pseudomonas koreensis* PgBE36	MH144259	99.00
		Pseudomonas fluorescens LBUM677	MG461479	99.00
**p9**	MK559913	*Pseudomonas koreensis* PgBE56	MH144278	98.92
		Pseudomonas fluorescens LBUM677	MG461479	98.92
**p10**	MK559914	*Pseudomonas koreensis* PgBE36	MH144259	99.00
		Pseudomonas fluorescens LBUM677	MG461479	99.00
**p11**	MK559915	*Pseudomonas koreensis* B16-237	MK072686	98.99
		Pseudomonas fluorescens LBUM677	MG461479	98.99
**p12**	MK559917	*Pseudomonas koreensis* PgBE36	MH144259	99.11
		Pseudomonas fluorescens LBUM677	MG461479	99.11
**p13**	MK559919	*Pseudomonas granadensis* PMK4	MH266421	99.93
		Pseudomonas fluorescens CREA-C16	CP017951	99.93
**p14**	MK559921	*Pseudomonas koreensis* PgBE56	MH144278	98.87

		Pseudomonas fluorescens LBUM677	MG461479	98.87
		Pseudomonas moraviensis I5-6	KU229982	98.87
**p15**	MK559922	*Pseudomonas koreensis* PgBE56	MH144278	98.85
		Pseudomonas fluorescens LBUM677	MG461479	98.85
		Pseudomonas moraviensis I5-6	KU229982	98.85
**p16**	MK559923	*Pseudomonas koreensis* B16-237	MK072686	99.07
		Pseudomonas fluorescens LBUM677	MG461479	99.07
**p17**	MK559924	*Pseudomonas granadensis* PMK4	MH266421	99.93
		Pseudomonas fluorescens CREA-C16	CP017951	99.93
**p18**	MK559925	*Pseudomonas koreensis* PgBE56	MH144278	98.99
		Pseudomonas fluorescens LBUM677	MG461479	98.99
		Pseudomonas moraviensis I5-6	KU229982	98.99
**p19**	MK559927	*Pseudomonas azotoformans* ICMP 14360	MK356438	99.93
		Pseudomonas libanensis DMSP-1	CP034425	99.93
		Pseudomonas gessardii 4G497	MG972901	99.93
**p20**	MK559929	*Pseudomonas koreensis* PgBE56	MH144278	98.92
		Pseudomonas fluorescens LBUM677	MG461479	98.92
		Pseudomonas moraviensis I5-6	KU229982	98.92
**p23**	MK559930	*Pseudomonas lutea* BG8	KJ997740	99.92
**p24**	MK559931	*Pseudomonas koreensis* PgBE56	MH144278	98.92
		Pseudomonas fluorescens LBUM677	MG461479	98.92
		Pseudomonas moraviensis I5-6	KU229982	98.92
**p25**	MK559932	*Pseudomonas granadensis* PMK4	MH266421	99.93
		Pseudomonas fluorescens CREA-C16	CP017951	99.93
**p30**	MK559933	*Pseudomonas granadensis* PMK4	MH266421	99.86
		Pseudomonas fluorescens CREA-C16	CP017951	99.86
		Pseudomonas reinekei SN21	KC790314	99.86
**p31**	MK559934	*Pseudomonas granadensis* PMK4	MH266421	99.93
		Pseudomonas fluorescens CREA-C16	CP017951	99.93
**p33**	MK559935	*Pseudomonas granadensis* PMK4	MH266421	99.92
		Pseudomonas fluorescens CREA-C16	CP017951	99.92
**p34**	MK559936	*Pseudomonas koreensis* F9-9	KT382238	99.00
**p36**	MK559937	*Pseudomonas granadensis* PMK4	MH266421	99.93
		Pseudomonas fluorescens CREA-C16	CP017951	99.93
**p37**	MK559938	*Pseudomonas granadensis* PMK4	MH266421	99.93
		Pseudomonas fluorescens CREA-C16	CP017951	99.93
**p42**	MK559939	*Pseudomonas azotoformans* ICMP 14360	MK356438	100.00
		Pseudomonas gessardii 4G497	MG972901	100.00
		Pseudomonas synxantha KGGI14	MH079449	100.00
		Pseudomonas fluorescens YPS3	MH580200	100.00
**p43**	MK559940	*Pseudomonas azotoformans* ICMP 14360	MK356438	100.00
		Pseudomonas gessardii 4G497	MG972901	100.00
		Pseudomonas fluorescens YPS3	MH580200	100.00

**p44**	MK559941	*Pseudomonas granadensis* PMK4	MH266421	100.00
		Pseudomonas fluorescens CREA-C16	CP017951	100.00
**p45**	MK559949	*Pseudomonas granadensis* PMK4	MH266421	99.93
		Pseudomonas fluorescens CREA-C16	CP017951	99.93
**p47**	MK559942	*Pseudomonas koreensis* F9-9	KT382238	98.93
		Pseudomonas fluorescens LBUM677	MG461479	98.93
**p48**	MK559943	*Pseudomonas granadensis* PMK4	MH266421	99.93
		Pseudomonas fluorescens CREA-C16	CP017951	99.93
**p49**	MK559944	*Pseudomonas koreensis* PgBE56	MH144278	98.94
		Pseudomonas fluorescens LBUM677	MG461479	98.94
		Pseudomonas moraviensis I5-6	KU229982	98.94
**p50**	MK559945	*Pseudomonas koreensis* B16-237	MK072686	98.90
		Pseudomonas fluorescens LBUM677	MG461479	98.90
		Pseudomonas moraviensis I5-6	KU229982	98.90
**p51**	MK559920	*Pseudomonas koreensis* B16-237	MK072686	99.06
		Pseudomonas fluorescens LBUM677	MG461479	99.06
		Pseudomonas moraviensis I5-6	KU229982	99.06
**p52**	MK559926	*Pseudomonas koreensis* PgBE36	MH144259	99.00
		Pseudomonas fluorescens LBUM677	MG461479	99.00
**p53**	MK559928	*Pseudomonas mandelii* IHB B 10205	KR233778	100.00
**p54**	MK559912	*Pseudomonas helmanticensis* 28D7	MG269632	99.86
		Pseudomonas fluorescens 2F9	KT695813	99.86
**p55**	MK559911	*Pseudomonas helmanticensis* 28D7	MG269632	99.86
		Pseudomonas fluorescens 2F9	KT695813	99.86
**p56**	MK559916	*Pseudomonas granadensis* PMK4	MH266421	99.86
		Pseudomonas fluorescens CREA-C16	CP017951	99.86
		Pseudomonas reinekei SN21	KC790314	99.86
**p57**	MK559918	*Pseudomonas koreensis* PgBE56	MH144278	98.92
		Pseudomonas fluorescens LBUM677	MG461479	98.92
		Pseudomonas moraviensis I5-6	KU229982	98.92
**p60**	MK559947	*Pseudomonas migulae* HRT13	JF778700	99.93
**p61**	MK559946	*Pseudomonas fluorescens* LBUM570	MG461471	99.93
		Pseudomonas helmanticensis 31A-P4	MF062636	99.93
**p62**	MK559948	*Pseudomonas koreensis* PgBE56	MH144278	98.94
		Pseudomonas fluorescens LBUM677	MG461479	98.94
		Pseudomonas moraviensis I5-6	KU229982	98.94
**AFG3.2**	KT314126	*Arthrobacter sulfureus* DSM 20167^{T}	NR_026237	99.28
**AFG7.2**	KT314127	*Arthrobacter phenanthrenivorans* Sphe3^{T}	NR_074770	99.42
**AFG8**	KT314103	*Arthrobacter nitroguajacolicus* G2-1^{T}	NR_027199	99.93
**AFG15.2**	KT314122	*Arthrobacter siccitolerans* 4J27^{T}	NR_108849	99.93
**AFG16.1**	KT314130	*Arthrobacter nitroguajacolicus* G2-1^{T}	NR_027199	99.93
**AFG17**	KT314108	*Arthrobacter siccitolerans* 4J27^{T}	NR_108849	99.49
**AFG17.2**	KT314152	*Arthrobacter globiformis* JCM 1332^{T}	NR_112192	99.71

**AFG19.3**	KT314147	*Arthrobacter globiformis* JCM 1332^T	NR_112192	99.71
**AFG20**	KT314111	*Arthrobacter globiformis* JCM 1332^T	NR_112192	99.57
**AFG20.1**	KT314142	*Arthrobacter globiformis* JCM 1332^T	NR_112192	99.49
**AFG22**	KT314113	*Arthrobacter nitroguajacolicus* G2-1^T	NR_027199	99.86
**AFG27.1**	KT314139	*Arthrobacter globiformis* JCM 1332^T	NR_112192	99.49
