# Supplementary material for: Bacteria from the endosphere and rhizosphere of Quercus spp. use mainly cell wall-associated enzymes to decompose organic matter
Source: PLoS One. 2019 Mar 25;14(3):e0214422. doi: 10.1371/journal.pone.0214422 (PMC6433265; doi:10.1371/journal.pone.0214422)
Supplement: S3 Table — Data represent means and standard deviations of activities of those enzymes produced by two or more strains. A ‘-’ indicates values below detection limit. An ‘*’ indicates values that were recorded just for one strain, the one which is shown in parentheses. Abbreviations of enzymes: bG: β-glucosidase; Pho: acid phosphatase; Lip: lipase; bM: β-mannosidase; aA: α-arabinosidase; bX: β-xylosidase; bGal: β-galactosidase; CBH: cellobiohydrolase; aG: α-glucosidase; ChTN: chitinase; aGal: α-galactosidase; bGlu: β-glucuronidase. For each bacterial genus, significant differences between the activity of those enzymes detected in both fractions were calculated by using the non-parametric Mann-Whitney U test at a confidence level of 95%. (PDF) [file pone.0214422.s003.pdf]

S3 Table. Activity of cell-wall bound and freely-released enzymes produced by genera *Luteibacter*, *Pseudomonas* and *Arthrobacter* isolated from the root endosphere or the rhizosphere of *Quercus* spp. trees. Data represent means and standard deviations of activities of those enzymes produced by two or more strains. A '-' indicates values below detection limit. An '*' indicates values that were recorded just for one strain, the one which is shown in parentheses. Abbreviations of enzymes: **bG**: β-glucosidase; **Pho**: acid phosphatase; **Lip**: lipase; **bM**: β-mannosidase; **aA**: α-arabinosidase; **bX**: β-xylosidase; **bGal**: β-galactosidase; **CBH**: cellobiohydrolase; **aG**: α-glucosidase; **ChTN**: chitinase; **aGal**: α-galactosidase; **bGlu**: β-glucuronidase. For each bacterial genus, significant differences between the activity of those enzymes detected in both fractions were calculated by using the non-parametric Mann-Whitney U test at a confidence level of 95%.

	**Activity** (nM min^{-1} mL^{-1})								
	Luteibacter			***Pseudomonas***			***Arthrobacter***		
	**Bound**	**Free**	**P value**	**Bound**	**Free**	**P value**	**Bound**	**Free**	**P value**
**bG**	4152 ± 250	17 ± 9	0.028	203 ± 297	104 ± 95	0.216	18^* (AFG15.2)	32 ± 16	
**Pho**	5240 ± 364	128 ± 58	$7.05 \cdot 10^{-7}$	2729 ± 2105	1172 ± 1300	$7.86 \cdot 10^{-6}$	57 ± 75	22 ± 8	0.490
**Lip**	5415 ± 343	3134 ± 468	$7.05 \cdot 10^{-7}$	5451 ± 907	3287 ± 1235	$8.96 \cdot 10^{-12}$	308 ± 325	316 ± 300	0.977
**bM**	874 ± 149	10^* (L50)		12^* (p23)	42 ± 44		-	-	
**aA**	26 ± 6	-		-	44 ± 68		14 ± 1	-	
**bX**	104 ± 14	-		30 ± 12	43 ± 46	0.896	11^* (AFG15.2)	28^* (AFG7.2)	
**bGal**	1163 ± 195	-		-	42 ± 54		188 ± 151	73 ± 87	0.105
**CBH**	130 ± 24	-		30^* (p23)	44 ± 47		-	-	
**aG**	594 ± 230	-		10^* (p20)	39 ± 44		-	66 ± 99	
**ChTN**	11 ± 1	-		-	44 ± 52		63 ± 8	13^* (AFG22)	
**aGal**	587 ± 92	-		-	124 ± 243		-	15 ± 4	
**bGlu**	-	-		-	41 ± 41		-	87 ± 154	
