# Supplementary material for: Bacteria from the endosphere and rhizosphere of Quercus spp. use mainly cell wall-associated enzymes to decompose organic matter
Source: PLoS One. 2019 Mar 25;14(3):e0214422. doi: 10.1371/journal.pone.0214422 (PMC6433265; doi:10.1371/journal.pone.0214422)
Supplement: S4 Table — Data represent means and standard deviations of the total activity of three replicates. Abbreviations of enzymes: bG: β-glucosidase; Pho: acid phosphatase; Lip: lipase; bM: β-mannosidase; aA: α-arabinosidase; bX: β-xylosidase; bGal: β-galactosidase; CBH: cellobiohydrolase; aG: α-glucosidase; ChTN: chitinase; aGal: α-galactosidase; bGlu: β-glucuronidase. A ‘-’ indicates values below detection limit (PDF) [file pone.0214422.s004.pdf]

S4 Table. Activity of cell-bound and freely-released enzymes, and total enzymatic activity of strains of genus *Luteibacter*. Data represent means and standard deviations of the total activity of three replicates. Abbreviations of enzymes: **bG**: β-glucosidase; **Pho**: acid phosphatase; **Lip**: lipase; **bM**: β-mannosidase; **aA**: α-arabinosidase; **bX**: β-xylosidase; **bGal**: β-galactosidase; **CBH**: cellobiohydrolase; **aG**: α-glucosidase; **ChTN**: chitinase; **aGal**: α-galactosidase; **bGlu**: β-glucuronidase. A '-' indicates values below detection limit.

**Strain:**	**L1**		
	**Total activity (nM min^{-1} mL^{-1})**	**Bound (%)**	**Free (%)**
**bG**	3905 ± 88	100	-
**Pho**	5208 ± 141	97	3
**Lip**	7823 ± 354	64	36
**bM**	675 ± 113	100	-
**aA**	16 ± 1	100	-
**bX**	82 ± 5	100	-
**bGal**	910 ± 23	100	-
**CBH**	98 ± 5	100	-
**aG**	377 ± 51	100	-
**ChTN**	-	-	-
**aGal**	421 ± 6	100	-
**bGlu**	-	-	-
	**Enzymes produced (%)**		**83.3**

**Strain:**	**L3**		
	**Total activity (nM min^{-1} mL^{-1})**	**Bound (%)**	**Free (%)**
**bG**	3832 ± 109	100	-
**Pho**	5013 ± 168	97	3
**Lip**	8021 ± 59	60	40
**bM**	848 ± 69	100	-
**aA**	28 ± 1	100	-
**bX**	105 ± 7	100	-
**bGal**	1174 ± 27	100	-
**CBH**	131 ± 4	100	-
**aG**	600 ± 82	100	-
**ChTN**	-	-	-
**aGal**	564 ± 4	100	-
**bGlu**	-	-	-
	**Enzymes produced (%)**		**83.3**

**Strain:**	**L5**		
	**Total activity (nM min^{-1} mL^{-1})**	**Bound (%)**	**Free (%)**
**bG**	4030 ± 147	100	-
**Pho**	5202 ± 201	98	2
**Lip**	8312 ± 53	61	39
**bM**	825 ± 37	100	-
**aA**	31 ± 1	100	-
**bX**	104 ± 9	100	-
**bGal**	1286 ± 50	100	-
**CBH**	146 ± 8	100	-
**aG**	977 ± 128	100	-
**ChTN**	11 ± 1	100	-
**aGal**	634 ± 5	100	-
**bGlu**	-	-	-
	**Enzymes produced (%)**		**91.7**

**Strain:**	**L6**		
	**Total activity (nM min^{-1} mL^{-1})**	**Bound (%)**	**Free (%)**
**bG**	4123 ± 293	100	-
**Pho**	5030 ± 272	99	1
**Lip**	8794 ± 201	61	39
**bM**	943 ± 137	100	-
**aA**	29 ± 1	100	-
**bX**	110 ± 12	100	-
**bGal**	1274 ± 56	100	-
**CBH**	146 ± 10	100	-
**aG**	875 ± 123	100	-
**ChTN**	-	-	-
**aGal**	639 ± 8	100	-
**bGlu**	-	-	-
	**Enzymes produced (%)**		**83.3**

**Strain:**	**L7**		
	**Total activity (nM min^{-1} mL^{-1})**	**Bound (%)**	**Free (%)**
**bG**	4039 ± 214	100	-
**Pho**	5668 ± 224	98	2
**Lip**	10003 ± 61	54	46
**bM**	880 ± 72	100	-
**aA**	32 ± 1	100	-
**bX**	107 ± 10	100	-
**bGal**	1349 ± 89	100	-
**CBH**	157 ± 13	100	-
**aG**	965 ± 113	100	-
**ChTN**	11 ± 1	100	-
**aGal**	619 ± 14	100	-
**bGlu**	-	-	-
	**Enzymes produced (%)**		**91.7**

**Strain:**	**L8**		
	**Total activity (nM min^{-1} mL^{-1})**	**Bound (%)**	**Free (%)**
**bG**	4042 ± 125	100	-
**Pho**	5110 ± 89	98	2
**Lip**	8643 ± 173	64	36
**bM**	905 ± 56	100	-
**aA**	27 ± 2	100	-
**bX**	115 ± 10	100	-
**bGal**	1239 ± 53	100	-
**CBH**	145 ± 8	100	-
**aG**	619 ± 49	100	-
**ChTN**	-	-	-
**aGal**	610 ± 1	100	-
**bGlu**	-	-	-
	**Enzymes produced (%)**		**83.3**

Strain:	L9		
	Total activity (nM min^{-1} mL^{-1})	Bound (%)	Free (%)
**bG**	4397 ± 209	100	-
**Pho**	5462 ± 216	98	2
**Lip**	8847 ± 72	66	34
**bM**	951 ± 79	100	-
**aA**	30 ± 1	100	-
**bX**	126 ± 17	100	-
**bGal**	1302 ± 77	100	-
**CBH**	156 ± 14	100	-
**aG**	738 ± 146	100	-
**ChTN**	11 ± 3	100	-
**aGal**	659 ± 7	100	-
**bGlu**	-	-	-
	**Enzymes produced (%)**		**91.7**

Strain:	L10		
	Total activity (nM min^{-1} mL^{-1})	Bound (%)	Free (%)
**bG**	4466 ± 158	100	-
**Pho**	5316 ± 153	98	2
**Lip**	8701 ± 111	64	36
**bM**	1002 ± 102	100	-
**aA**	29 ± 0	100	-
**bX**	129 ± 16	100	-
**bGal**	1277 ± 71	100	-
**CBH**	157 ± 14	100	-
**aG**	747 ± 113	100	-
**ChTN**	13 ± 1	100	-
**aGal**	671 ± 4	100	-
**bGlu**	-	-	-
	**Enzymes produced (%)**		**91.7**

Strain:	L12		
	Total activity (nM min^{-1} mL^{-1})	Bound (%)	Free (%)
**bG**	4031 ± 86	100	-
**Pho**	4988 ± 51	97	3
**Lip**	8160 ± 94	63	37
**bM**	877 ± 126	100	-
**aA**	21 ± 1	100	-
**bX**	96 ± 8	100	-
**bGal**	986 ± 41	100	-
**CBH**	117 ± 6	100	-
**aG**	477 ± 88	100	-
**ChTN**	-	-	-
**aGal**	554 ± 4	100	-
**bGlu**	-	-	-
	**Enzymes produced (%)**		**83.3**

Strain:	L13		
	Total activity (nM min^{-1} mL^{-1})	Bound (%)	Free (%)
**bG**	4004 ± 58	100	-
**Pho**	4883 ± 17	98	2
**Lip**	7757 ± 235	67	33
**bM**	850 ± 111	100	-
**aA**	22 ± 1	100	-
**bX**	94 ± 8	100	-
**bGal**	955 ± 30	100	-
**CBH**	110 ± 6	100	-
**aG**	446 ± 83	100	-
**ChTN**	-	-	-
**aGal**	543 ± 8	100	-
**bGlu**	-	-	-
	**Enzymes produced (%)**		**83.3**

Strain:	L14		
	Total activity (nM min^{-1} mL^{-1})	Bound (%)	Free (%)
**bG**	4743 ± 168	99	1
**Pho**	6305 ± 165	97	3
**Lip**	8741 ± 45	66	34
**bM**	1238 ± 162	100	-
**aA**	-	-	-
**bX**	82 ± 7	100	-
**bGal**	1329 ± 48	100	-
**CBH**	116 ± 11	100	-
**aG**	611 ± 34	100	-
**ChTN**	-	-	-
**aGal**	716 ± 13	100	-
**bGlu**	-	-	-
	**Enzymes produced (%)**		**75**

Strain:	L15		
	Total activity (nM min^{-1} mL^{-1})	Bound (%)	Free (%)
**bG**	3856 ± 38	99	1
**Pho**	5267 ± 89	95	5
**Lip**	8574 ± 159	59	41
**bM**	610 ± 54	100	-
**aA**	18 ± 2	100	-
**bX**	91 ± 7	100	-
**bGal**	848 ± 14	100	-
**CBH**	96 ± 4	100	-
**aG**	267 ± 29	100	-
**ChTN**	-	-	-
**aGal**	409 ± 9	100	-
**bGlu**	-	-	-
	**Enzymes produced (%)**		**83.3**

Strain:	L18		
	Total activity (nM min^{-1} mL^{-1})	Bound (%)	Free (%)
bG	4141 ± 90	100	-
Pho	5245 ± 30	99	1
Lip	8037 ± 190	66	34
bM	798 ± 102	100	-
aA	20 ± 2	100	-
bX	98 ± 10	100	-
bGal	990 ± 40	100	-
CBH	107 ± 6	100	-
aG	363 ± 55	100	-
ChTN	-	-	-
aGal	512 ± 4	100	-
bGlu	-	-	-
	Enzymes produced (%)		83.3

Strain:	L20		
	Total activity (nM min^{-1} mL^{-1})	Bound (%)	Free (%)
bG	4151 ± 88	100	-
Pho	5140 ± 52	99	1
Lip	7971 ± 217	67	33
bM	640 ± 65	100	-
aA	19 ± 1	100	-
bX	94 ± 9	100	-
bGal	893 ± 52	100	-
CBH	103 ± 9	100	-
aG	339 ± 73	100	-
ChTN	10 ± 1	100	-
aGal	460 ± 0	100	-
bGlu	-	-	-
	Enzymes produced (%)		91.7

Strain:	L21		
	Total activity (nM min^{-1} mL^{-1})	Bound (%)	Free (%)
bG	4535 ± 225	100	-
Pho	5932 ± 169	97	3
Lip	9078 ± 129	66	34
bM	991 ± 84	100	-
aA	32 ± 1	100	-
bX	121 ± 16	100	-
bGal	1407. ± 77	100	-
CBH	164 ± 9	100	-
aG	831 ± 276	100	-
ChTN	-	-	-
aGal	687 ± 7	100	-
bGlu	-	-	-
	Enzymes produced (%)		83.3

Strain:	L22		
	Total activity (nM min^{-1} mL^{-1})	Bound (%)	Free (%)
bG	4032 ± 149	100	-
Pho	5647 ± 81	97	3
Lip	8729 ± 174	68	32
bM	884 ± 55	100	-
aA	25 ± 1	100	-
bX	96 ± 11	100	-
bGal	1115 ± 55	100	-
CBH	116 ± 10	100	-
aG	329 ± 78	100	-
ChTN	-	-	-
aGal	605 ± 2	100	-
bGlu	-	-	-
	Enzymes produced (%)		83.3

Strain:	L50		
	Total activity (nM min^{-1} mL^{-1})	Bound (%)	Free (%)
bG	4297 ± 184	100	-
Pho	5839 ± 91	98	2
Lip	9143 ± 68	62	38
bM	959 ± 49	99	1
aA	38 ± 0	100	-
bX	118 ± 7	100	-
bGal	1442 ± 49	100	-
CBH	149 ± 19	100	-
aG	541 ± 2	100	-
ChTN	11 ± 3	100	-
aGal	668 ± 4	100	-
bGlu	-	-	-
	Enzymes produced (%)		91.7
