# Supplementary material for: Bacteria from the endosphere and rhizosphere of Quercus spp. use mainly cell wall-associated enzymes to decompose organic matter
Source: PLoS One. 2019 Mar 25;14(3):e0214422. doi: 10.1371/journal.pone.0214422 (PMC6433265; doi:10.1371/journal.pone.0214422)
Supplement: S5 Table — Data represent means and standard deviations of the total activity of three replicates. Abbreviations of enzymes: bG: β-glucosidase; Pho: acid phosphatase; Lip: lipase; bM: β-mannosidase; aA: α-arabinosidase; bX: β-xylosidase; bGal: β-galactosidase; CBH: cellobiohydrolase; aG: α-glucosidase; ChTN: chitinase; aGal: α-galactosidase; bGlu: β-glucuronidase. A ‘-’ indicates values below detection limit. (PDF) [file pone.0214422.s005.pdf]

S5 Table. Activity of cell-bound and freely-released enzymes, and total enzymatic activity of strains of genus *Pseudomonas*. Data represent means and standard deviations of the total activity of three replicates. Abbreviations of enzymes: **bG**: β-glucosidase; **Pho**: acid phosphatase; **Lip**: lipase; **bM**: β-mannosidase; **aA**: α-arabinosidase; **bX**: β-xylosidase; **bGal**: β-galactosidase; **CBH**: cellobiohydrolase; **aG**: α-glucosidase; **ChTN**: chitinase; **aGal**: α-galactosidase; **bGlu**: β-glucuronidase. A '-' indicates values below detection limit.

Strain:	p1			Strain:	p2			Strain:	p3		
	Total activity (nM min^{-1} mL^{-1})	Bound (%)	Free (%)		Total activity (nM min^{-1} mL^{-1})	Bound (%)	Free (%)		Total activity (nM min^{-1} mL^{-1})	Bound (%)	Free (%)
**bG**	327 ± 4	75	25	**bG**	-	-	-	**bG**	302 ± 3	53	47
**Pho**	7242 ± 108	58	42	**Pho**	4970 ± 149	97	3	**Pho**	5750 ± 88	52	48
**Lip**	7880 ± 105	68	32	**Lip**	8213 ± 81	64	36	**Lip**	8846 ± 100	67	33
**bM**	-	-	-	**bM**	-	-	-	**bM**	-	-	-
**aA**	-	-	-	**aA**	-	-	-	**aA**	-	-	-
**bX**	-	-	-	**bX**	-	-	-	**bX**	-	-	-
**bGal**	-	-	-	**bGal**	-	-	-	**bGal**	-	-	-
**CBH**	-	-	-	**CBH**	-	-	-	**CBH**	-	-	-
**aG**	-	-	-	**aG**	-	-	-	**aG**	-	-	-
**ChTN**	-	-	-	**ChTN**	-	-	-	**ChTN**	-	-	-
**aGal**	-	-	-	**aGal**	15 ± 1	-	100.00	**aGal**	-	-	-
**bGlu**	-	-	-	**bGlu**	-			**bGlu**	-	-	-
	**Enzymes produced (%)**		**25**		**Enzymes produced (%)**		**25**		**Enzymes produced (%)**		**25**

Strain:	p4			Strain:	p7			Strain:	p9		
	Total activity (nM min^{-1} mL^{-1})	Bound (%)	Free (%)		Total activity (nM min^{-1} mL^{-1})	Bound (%)	Free (%)		Total activity (nM min^{-1} mL^{-1})	Bound (%)	Free (%)
**bG**	125 ± 4	11	89	**bG**	-	-	-	**bG**	94 ± 6	61	39
**Pho**	2297 ± 65.	17	83	**Pho**	1647 ± 77	94	6	**Pho**	3521 ± 157	65	35
**Lip**	2938 ± 278	32	68	**Lip**	8681 ± 117	58	42	**Lip**	9614 ± 141	68	32
**bM**	-	-	-	**bM**	-	-	-	**bM**	17 ± 3	-	100
**aA**	-	-	-	**aA**	-	-	-	**aA**	14 ± 1	-	100
**bX**	-	-	-	**bX**	-	-	-	**bX**	16 ± 3	-	100
**bGal**	-	-	-	**bGal**	-	-	-	**bGal**	13 ± 3	-	100
**CBH**	-	-	-	**CBH**	-	-	-	**CBH**	13 ± 1	-	100
**aG**	-	-	-	**aG**	-	-	-	**aG**	20 ± 3	-	100
**ChTN**	-	-	-	**ChTN**	-	-	-	**ChTN**	-	-	-
**aGal**	760 ± 22	-	100	**aGal**	-	-	-	**aGal**	22 ± 1	-	100
**bGlu**	-	-	-	**bGlu**	-	-	-	**bGlu**	17 ± 2	-	100
	**Enzymes produced (%)**		**33.3**		**Enzymes produced (%)**		**16.7**		**Enzymes produced (%)**		**91.7**

Strain:	p10		
	Total activity (nM min^{-1} mL^{-1})	Bound (%)	Free (%)
bG	46 ± 2	100	-
Pho	6566 ± 138	58	42
Lip	10838 ± 355	53	47
bM	-	-	-
aA	-	-	-
bX	-	-	-
bGal	-	-	-
CBH	-	-	-
aG	-	-	-
ChTN	-	-	-
aGal	-	-	-
bGlu	-	-	-
Enzymes produced (%)			25

Strain:	p11		
	Total activity (nM min^{-1} mL^{-1})	Bound (%)	Free (%)
bG	160 ± 4	56	44
Pho	6440 ± 94	37	63
Lip	10667 ± 361	53	47
bM	-	-	-
aA	-	-	-
bX	-	-	-
bGal	-	-	-
CBH	-	-	-
aG	-	-	-
ChTN	-	-	-
aGal	-	-	-
bGlu	-	-	-
Enzymes produced (%)			25

Strain:	p12		
	Total activity (nM min^{-1} mL^{-1})	Bound (%)	Free (%)
bG	132 ± 2	58	42
Pho	5134 ± 148	40	60
Lip	8715 ± 108	57	43
bM	-	-	-
aA	-	-	-
bX	-	-	-
bGal	-	-	-
CBH	-	-	-
aG	-	-	-
ChTN	-	-	-
aGal	-	-	-
bGlu	-	-	-
Enzymes produced (%)			25

Strain:	p13		
	Total activity (nM min^{-1} mL^{-1})	Bound (%)	Free (%)
bG	-	-	-
Pho	276 ± 28	89	11
Lip	7327 ± 522	75	25
bM	-	-	-
aA	-	-	-
bX	-	-	-
bGal	-	-	-
CBH	-	-	-
aG	-	-	-
ChTN	-	-	-
aGal	-	-	-
bGlu	-	-	-
Enzymes produced (%)			16.7

Strain:	p14		
	Total activity (nM min^{-1} mL^{-1})	Bound (%)	Free (%)
bG	51 ± 3	100	-
Pho	6683 ± 215	61	39
Lip	10544 ± 617	55	45
bM	-	-	-
aA	-	-	-
bX	-	-	-
bGal	-	-	-
CBH	-	-	-
aG	-	-	-
ChTN	-	-	-
aGal	-	-	-
bGlu	-	-	-
Enzymes produced (%)			25

Strain:	p15		
	Total activity (nM min^{-1} mL^{-1})	Bound (%)	Free (%)
bG	141 ± 5	74	26
Pho	4375 ± 199	78	22
Lip	8101 ± 529	75	25
bM	-	-	-
aA	-	-	-
bX	-	-	-
bGal	-	-	-
CBH	-	-	-
aG	-	-	-
ChTN	-	-	-
aGal	-	-	-
bGlu	-	-	-
Enzymes produced (%)			25

Strain:	p16		
	Total activity (nM min^{-1} mL^{-1})	Bound (%)	Free (%)
bG	289 ± 9	49	51
Pho	7558 ± 281	55	45
Lip	9078 ± 509	68	32
bM	-	-	-
aA	-	-	-
bX	-	-	-
bGal	-	-	-
CBH	-	-	-
aG	-	-	-
ChTN	-	-	-
aGal	-	-	-
bGlu	-	-	-
Enzymes produced (%)			25

Strain:	p17		
	Total activity (nM min^{-1} mL^{-1})	Bound (%)	Free (%)
bG	-	-	-
Pho	261 ± 17	74	26
Lip	10518 ± 273	55	45
bM	-	-	-
aA	-	-	-
bX	19. ± 12	-	100
bGal	-	-	-
CBH	-	-	-
aG	20 ± 20	-	100
ChTN	-	-	-
aGal	-	-	-
bGlu	-	-	-
Enzymes produced (%)			33.3

Strain:	p18		
	Total activity (nM min^{-1} mL^{-1})	Bound (%)	Free (%)
bG	153 ± 45	83	17
Pho	7055 ± 355	76	24
Lip	7990 ± 108	82	18
bM	10 ± 5	-	100
aA	15 ± 4	-	100
bX	-	-	-
bGal	11 ± 1	-	100
CBH	-	-	-
aG	15 ± 1	-	100
ChTN	-	-	-
aGal	18 ± 2	-	100
bGlu	11 ± 1	-	100
Enzymes produced (%)			75

Strain:	p19		
	Total activity (nM min^{-1} mL^{-1})	Bound (%)	Free (%)
bG	-	-	-
Pho	1862 ± 55	96	4
Lip	8333 ± 157	62	38
bM	-	-	-
aA	-	-	-
bX	-	-	-
bGal	-	-	-
CBH	-	-	-
aG	-	-	-
ChTN	-	-	-
aGal	-	-	-
bGlu	-	-	-
Enzymes produced (%)			16.7

Strain:	p20		
	Total activity (nM min^{-1} mL^{-1})	Bound (%)	Free (%)
bG	1306 ± 27	71	29
Pho	8874 ± 127	63	37
Lip	8768 ± 325	67	33
bM	41 ± 5	-	100
aA	39 ± 2	-	100
bX	91 ± 6	42	58
bGal	36 ± 3	-	100
CBH	41 ± 3	-	100
aG	57 ± 4	18	82
ChTN	39 ± 1	-	100
aGal	43 ± 1	-	100
bGlu	42 ± 1	-	100
Enzymes produced (%)			100

Strain:	p23		
	Total activity (nM min^{-1} mL^{-1})	Bound (%)	Free (%)
bG	1449 ± 26	84	16
Pho	5676 ± 37	92	8
Lip	7270 ± 431	72	28
bM	150 ± 8	8	92
aA	210 ± 9	-	100
bX	151 ± 15	-	100
bGal	164 ± 0	-	100
CBH	167 ± 25	18	82
aG	154 ± 28	-	100
ChTN	148 ± 23	-	100
aGal	169 ± 7	-	100
bGlu	130 ± 11	-	100
Enzymes produced (%)			100

Strain:	p24		
	Total activity (nM min^{-1} mL^{-1})	Bound (%)	Free (%)
bG	602 ± 31	67	33
Pho	8586 ± 231	67	33
Lip	10074 ± 104	66	34
bM	-	-	-
aA	-	-	-
bX	39 ± 3	57	43
bGal	-	-	-
CBH	-	-	-
aG	11 ± 3	-	100
ChTN	-	-	-
aGal	-	-	-
bGlu	-	-	-
Enzymes produced (%)			41.7

Strain:	p25		
	Total activity (nM min^{-1} mL^{-1})	Bound (%)	Free (%)
bG	-	-	-
Pho	175 ± 7	77	23
Lip	9245 ± 179	53	47
bM	-	-	-
aA	-	-	-
bX	-	-	-
bGal	-	-	-
CBH	-	-	-
aG	-	-	-
ChTN	-	-	-
aGal	-	-	-
bGlu	-	-	-
Enzymes produced (%)			16.7

Strain:	p30		
	Total activity (nM min^{-1} mL^{-1})	Bound (%)	Free (%)
bG	-	-	-
Pho	210 ± 12	83	17
Lip	9038 ± 166	52	48
bM	-	-	-
aA	-	-	-
bX	-	-	-
bGal	-	-	-
CBH	-	-	-
aG	-	-	-
ChTN	-	-	-
aGal	-	-	-
bGlu	-	-	-
Enzymes produced (%)			16.7

Strain:	p31		
	Total activity (nM min^{-1} mL^{-1})	Bound (%)	Free (%)
bG	-	-	-
Pho	234 ± 6	75	25
Lip	10589 ± 350	46	54
bM	-	-	-
aA	-	-	-
bX	-	-	-
bGal	-	-	-
CBH	-	-	-
aG	-	-	-
ChTN	-	-	-
aGal	-	-	-
bGlu	-	-	-
Enzymes produced (%)			16.7

Strain:	p33		
	Total activity (nM min^{-1} mL^{-1})	Bound (%)	Free (%)
bG	-	-	-
Pho	262 ± 7	76	24
Lip	8217 ± 106	66	34
bM	-	-	-
aA	-	-	-
bX	-	-	-
bGal	-	-	-
CBH	-	-	-
aG	-	-	-
ChTN	-	-	-
aGal	-	-	-
bGlu	-	-	-
Enzymes produced (%)			16.7

Strain:	p34		
	Total activity (nM min^{-1} mL^{-1})	Bound (%)	Free (%)
bG	194 ± 9	81	19
Pho	5852 ± 134	87	13
Lip	8680 ± 65	75	25
bM	21 ± 3	-	100
aA	16 ± 3	-	100
bX	20 ± 4	-	100
bGal	19 ± 1	-	100
CBH	14 ± 1	-	100
aG	23 ± 2	-	100
ChTN	14 ± 3	-	100
aGal	25 ± 6	-	100
bGlu	21 ± 5	-	100
Enzymes produced (%)			100

Strain:	p36		
	Total activity (nM min^{-1} mL^{-1})	Bound (%)	Free (%)
bG	-	-	-
Pho	274 ± 11	82	18
Lip	9420 ± 130	56	44
bM	-	-	-
aA	-	-	-
bX	-	-	-
bGal	-	-	-
CBH	-	-	-
aG	-	-	-
ChTN	-	-	-
aGal	-	-	-
bGlu	-	-	-
Enzymes produced (%)			16.7

Strain:	p37		
	Total activity (nM min^{-1} mL^{-1})	Bound (%)	Free (%)
bG	-	-	-
Pho	209 ± 16	82	18
Lip	8519 ± 485	55	45
bM	-	-	-
aA	-	-	-
bX	-	-	-
bGal	-	-	-
CBH	-	-	-
aG	-	-	-
ChTN	-	-	-
aGal	-	-	-
bGlu	-	-	-
Enzymes produced (%)			16.7

Strain:	p42		
	Total activity (nM min^{-1} mL^{-1})	Bound (%)	Free (%)
bG	-	-	-
Pho	287 ± 16	90	10
Lip	8321 ± 176	69	31
bM	-	-	-
aA	-	-	-
bX	-	-	-
bGal	-	-	-
CBH	-	-	-
aG	-	-	-
ChTN	-	-	-
aGal	-	-	-
bGlu	-	-	-
Enzymes produced (%)			16.7

Strain:	p43		
	Total activity (nM min^{-1} mL^{-1})	Bound (%)	Free (%)
bG	-	-	-
Pho	430 ± 27	88	12
Lip	10696 ± 152	56	44
bM	-	-	-
aA	-	-	-
bX	-	-	-
bGal	-	-	-
CBH	-	-	-
aG	-	-	-
ChTN	-	-	-
aGal	-	-	-
bGlu	-	-	-
Enzymes produced (%)			16.7

Strain:	p44		
	Total activity (nM min^{-1} mL^{-1})	Bound (%)	Free (%)
bG	-	-	-
Pho	313 ± 15	74	26
Lip	10587 ± 360	49	51
bM	-	-	-
aA	-	-	-
bX	-	-	-
bGal	-	-	-
CBH	-	-	-
aG	-	-	-
ChTN	-	-	-
aGal	-	-	-
bGlu	-	-	-
Enzymes produced (%)			16.7

Strain:	p45		
	Total activity (nM min^{-1} mL^{-1})	Bound (%)	Free (%)
bG	-	-	-
Pho	343 ± 19	75	25
Lip	8891 ± 274	66	34
bM	-	-	-
aA	-	-	-
bX	-	-	-
bGal	-	-	-
CBH	-	-	-
aG	-	-	-
ChTN	-	-	-
aGal	-	-	-
bGlu	-	-	-
Enzymes produced (%)			16.7

Strain:	p47		
	Total activity (nM min^{-1} mL^{-1})	Bound (%)	Free (%)
bG	226 ± 9	77	23
Pho	6384 ± 132	63	37
Lip	8911 ± 107	66	34
bM	-	-	-
aA	-	-	-
bX	-	-	-
bGal	-	-	-
CBH	-	-	-
aG	-	-	-
ChTN	-	-	-
aGal	-	-	-
bGlu	-	-	-
Enzymes produced (%)			25

Strain:	p48		
	Total activity (nM min^{-1} mL^{-1})	Bound (%)	Free (%)
bG	-	-	-
Pho	327 ± 17	77	23
Lip	11059 ± 232	46	54
bM	-	-	-
aA	-	-	-
bX	-	-	-
bGal	-	-	-
CBH	-	-	-
aG	-	-	-
ChTN	-	-	-
aGal	-	-	-
bGlu	-	-	-
Enzymes produced (%)			16.7

Strain:	p49		
	Total activity (nM min^{-1} mL^{-1})	Bound (%)	Free (%)
bG	99 ± 3	47	53
Pho	6534 ± 72	76	24
Lip	9598 ± 175	67	33
bM	41 ± 9	-	100
aA	28 ± 2	-	100
bX	40 ± 2	-	100
bGal	32 ± 4	-	100
CBH	39 ± 5	-	100
aG	32 ± 4	-	100
ChTN	29 ± 4	-	100
aGal	30 ± 7	-	100
bGlu	39 ± 2	-	100
Enzymes produced (%)			100

Strain:	p50		
	Total activity (nM min^{-1} mL^{-1})	Bound (%)	Free (%)
bG	335 ± 9	50	50
Pho	8027 ± 253	63	37
Lip	8534 ± 169	71	29
bM	-	-	-
aA	-	-	-
bX	-	-	-
bGal	-	-	-
CBH	-	-	-
aG	-	-	-
ChTN	-	-	-
aGal	-	-	-
bGlu	-	-	-
Enzymes produced (%)			25

Strain:	p51		
	Total activity (nM min^{-1} mL^{-1})	Bound (%)	Free (%)
bG	59 ± 5	100	-
Pho	6769 ± 128	76	24
Lip	6985 ± 47	73	27
bM	-	-	-
aA	-	-	-
bX	-	-	-
bGal	-	-	-
CBH	-	-	-
aG	-	-	-
ChTN	-	-	-
aGal	-	-	-
bGlu	-	-	-
Enzymes produced (%)			25

Strain:	p52		
	Total activity (nM min^{-1} mL^{-1})	Bound (%)	Free (%)
bG	113 ± 11	100	-
Pho	7756 ± 174	72	28
Lip	7292 ± 193	80	20
bM	-	-	-
aA	-	-	-
bX	-	-	-
bGal	-	-	-
CBH	-	-	-
aG	-	-	-
ChTN	-	-	-
aGal	-	-	-
bGlu	-	-	-
Enzymes produced (%)			25

Strain:	p53		
	Total activity (nM min^{-1} mL^{-1})	Bound (%)	Free (%)
bG	-	-	-
Pho	4719 ± 217	98	2
Lip	8094 ± 365	66	34
bM	-	-	-
aA	-	-	-
bX	-	-	-
bGal	-	-	-
CBH	-	-	-
aG	-	-	-
ChTN	-	-	-
aGal	-	-	-
bGlu	-	-	-
Enzymes produced (%)			16.7

Strain:	p54		
	Total activity (nM min^{-1} mL^{-1})	Bound (%)	Free (%)
bG	21 ± 3	-	100
Pho	5460 ± 106	98	2
Lip	7650 ± 241	80	20
bM	29 ± 3	-	100
aA	21 ± 3	-	100
bX	25 ± 1	-	100
bGal	22 ± 3	-	100
CBH	20 ± 3	-	100
aG	31 ± 2	-	100
ChTN	23 ± 2	-	100
aGal	31 ± 3	-	100
bGlu	28 ± 4	-	100
Enzymes produced (%)			100

Strain:	p55		
	Total activity (nM min^{-1} mL^{-1})	Bound (%)	Free (%)
bG	-	-	-
Pho	2468 ± 332	99	1
Lip	7899 ± 468	66	34
bM	-	-	-
aA	-	-	-
bX	-	-	-
bGal	-	-	-
CBH	-	-	-
aG	-	-	-
ChTN	-	-	-
aGal	-	-	-
bGlu	-	-	-
Enzymes produced (%)			16.7

Strain:	p56		
	Total activity (nM min^{-1} mL^{-1})	Bound (%)	Free (%)
bG	-	-	-
Pho	253 ± 16	90	10
Lip	9641 ± 616	51	49
bM	-	-	-
aA	-	-	-
bX	-	-	-
bGal	-	-	-
CBH	-	-	-
aG	-	-	-
ChTN	-	-	-
aGal	-	-	-
bGlu	-	-	-
Enzymes produced (%)			16.7

Strain:	p57		
	Total activity (nM min^{-1} mL^{-1})	Bound (%)	Free (%)
bG	46 ± 7	63	37
Pho	4855 ± 154	42	58
Lip	7765 ± 86	50	50
bM	-	-	-
aA	-	-	-
bX	-	-	-
bGal	-	-	-
CBH	-	-	-
aG	-	-	-
ChTN	-	-	-
aGal	-	-	-
bGlu	-	-	-
Enzymes produced (%)			25

Strain:	p60		
	Total activity (nM min^{-1} mL^{-1})	Bound (%)	Free (%)
bG	-	-	-
Pho	5468 ± 258	98	2
Lip	6737 ± 697	77	23
bM	-	-	-
aA	11 ± 4	-	100
bX	-	-	-
bGal	-	-	-
CBH	-	-	-
aG	-	-	-
ChTN	-	-	-
aGal	-	-	-
bGlu	-	-	-
Enzymes produced (%)			25

Strain:	p61			Strain:	p62		
	Total activity (nM min^{-1} mL^{-1})	Bound (%)	Free (%)		Total activity (nM min^{-1} mL^{-1})	Bound (%)	Free (%)
**bG**	-	-	-	**bG**	55 ± 1	100	-
**Pho**	3688 ± 199	99	1	**Pho**	5583 ± 90	69	31
**Lip**	9970 ± 641	56	44	**Lip**	7709 ± 345	75	25
**bM**	-	-	-	**bM**	-	-	-
**aA**	-	-	-	**aA**	-	-	-
**bX**	-	-	-	**bX**	-	-	-
**bGal**	-	-	-	**bGal**	-	-	-
**CBH**	-	-	-	**CBH**	-	-	-
**aG**	-	-	-	**aG**	-	-	-
**ChTN**	-	-	-	**ChTN**	-	-	-
**aGal**	-	-	-	**aGal**	-	-	-
**bGlu**	-	-	-	**bGlu**	-	-	-
	**Enzymes produced (%)**		**16.7**		**Enzymes produced (%)**		**25**
