# Supplementary material for: Bacteria from the endosphere and rhizosphere of Quercus spp. use mainly cell wall-associated enzymes to decompose organic matter
Source: PLoS One. 2019 Mar 25;14(3):e0214422. doi: 10.1371/journal.pone.0214422 (PMC6433265; doi:10.1371/journal.pone.0214422)
Supplement: S6 Table — Data represent means and standard deviations of the total activity of three replicates. Abbreviations of enzymes: bG: β-glucosidase; Pho: acid phosphatase; Lip: lipase; bM: β-mannosidase; aA: α-arabinosidase; bX: β-xylosidase; bGal: β-galactosidase; CBH: cellobiohydrolase; aG: α-glucosidase; ChTN: chitinase; aGal: α-galactosidase; bGlu: β-glucuronidase. A ‘-’ indicates values below detection limit. (PDF) [file pone.0214422.s006.pdf]

S6 Table. Activity of cell-bound and freely-released enzymes, and total enzymatic activity of strains of genus *Arthrobacter*. Data represent means and standard deviations of the total activity of three replicates. Abbreviations of enzymes: **bG:** β-glucosidase; **Pho**: acid phosphatase; **Lip**: lipase; **bM**: β-mannosidase; **aA**: α-arabinosidase; **bX**: β-xylosidase; **bGal**: β-galactosidase; **CBH**: cellobiohydrolase; **aG**: α-glucosidase; **ChTN**: chitinase; **aGal**: α-galactosidase; **bGlu**: β-glucuronidase. A '-' indicates values below detection limit.

**Strain:**	**AFG3.2**			**Strain:**	**AFG7.2**			**Strain:**	**AFG8**		
	**Total activity (nM min^{-1} mL^{-1})**	**Bound (%)**	**Free (%)**		**Total activity (nM min^{-1} mL^{-1})**	**Bound (%)**	**Free (%)**		**Total activity (nM min^{-1} mL^{-1})**	**Bound (%)**	**Free (%)**
**bG**	20 ± 0	-	100	**bG**	-	-	-	**bG**	-	-	-
**Pho**	20 ± 2	100	-	**Pho**	148 ± 11	100	-	**Pho**	27 ± 8	100	-
**Lip**	906 ± 37	5	95	**Lip**	503 ± 26	63	37	**Lip**	1124 ± 62	33	67
**bM**	-	-	-	**bM**	-	-	-	**bM**	-	-	-
**aA**	-	-	-	**aA**	-	-	-	**aA**	-	-	-
**bX**	-	-	-	**bX**	28 ± 2	-	100	**bX**	-	-	-
**bGal**	-	-	-	**bGal**	286 ± 8	-	100	**bGal**	96 ± 7	-	100
**CBH**	-	-	-	**CBH**	-	-	-	**CBH**	-	-	-
**aG**	-	-	-	**aG**	-	-	-	**aG**	15 ± 2	-	100
**ChTN**	-	-	-	**ChTN**	-	-	-	**ChTN**	64 ± 2	100	-
**aGal**	-	-	-	**aGal**	-	-	-	**aGal**	12 ± 1	-	100
**bGlu**	400 ± 18	-	100	**bGlu**	39 ± 9	-	100	**bGlu**	29 ± 6	-	100
	**Enzymes produced (%)**		**33.3**		**Enzymes produced (%)**		**41.7**		**Enzymes produced (%)**		**58.3**

**Strain:**	**AFG15.2**			**Strain:**	**AFG16.1**			**Strain:**	**AFG17**		
	**Total activity (nM min^{-1} mL^{-1})**	**Bound (%)**	**Free (%)**		**Total activity (nM min^{-1} mL^{-1})**	**Bound (%)**	**Free (%)**		**Total activity (nM min^{-1} mL^{-1})**	**Bound (%)**	**Free (%)**
**bG**	18 ± 1	100	-	**bG**	-	-	-	**bG**	-	-	-
**Pho**	251 ± 16	100	-	**Pho**	50 ± 8	44	56	**Pho**	52 ± 5	71	29
**Lip**	1289 ± 63	95	5	**Lip**	1159 ± 60	31	69	**Lip**	535 ± 24	65	35
**bM**	-	-	-	**bM**	-	-	-	**bM**	-	-	-
**aA**	15 ± 0	100	-	**aA**	-	-	-	**aA**	-	-	-
**bX**	11 ± 1	100	-	**bX**	-	-	-	**bX**	-	-	-
**bGal**	122 ± 3	89	11	**bGal**	101 ± 7	-	100	**bGal**	63 ± 1	-	100
**CBH**	-	-	-	**CBH**	-	-	-	**CBH**	-	-	-
**aG**	-	-	-	**aG**	-	-	-	**aG**	-	-	-
**ChTN**	-	-	-	**ChTN**	54 ± 5	100	-	**ChTN**	-	-	-
**aGal**	17 ± 1	-	100	**aGal**	-	-	-	**aGal**	-	-	-
**bGlu**	-	-	-	**bGlu**	17 ± 1	-	100	**bGlu**	-	-	-
	**Enzymes produced (%)**		**58.3**		**Enzymes produced (%)**		**41.7**		**Enzymes produced (%)**		**25**

Strain:	AFG17.2		
	Total activity ($nM\ min^{-1}\ mL^{-1}$)	Bound (%)	Free (%)
bG	-	-	-
Pho	21 ± 6	100	-
Lip	168 ± 33	49	51
bM	-	-	-
aA	-	-	-
bX	-	-	-
bGal	-	-	-
CBH	-	-	-
aG	12 ± 1	-	100
ChTN	-	-	-
aGal	-	-	-
bGlu	-	-	-
	Enzymes produced (%)		25

Strain:	AFG19.3		
	Total activity ($nM\ min^{-1}\ mL^{-1}$)	Bound (%)	Free (%)
bG	-	-	-
Pho	29 ± 7	100	-
Lip	276 ± 34	39	61
bM	-	-	-
aA	-	-	-
bX	-	-	-
bGal	95 ± 3	49	51
CBH	-	-	-
aG	22 ± 2	-	100
ChTN	-	-	-
aGal	-	-	-
bGlu	-	-	-
	Enzymes produced (%)		33.3

Strain:	AFG20		
	Total activity ($nM\ min^{-1}\ mL^{-1}$)	Bound (%)	Free (%)
bG	-	-	-
Pho	-	-	-
Lip	238 ± 1	46	54
bM	-	-	-
aA	-	-	-
bX	-	-	-
bGal	-	-	-
CBH	-	-	-
aG	-	-	-
ChTN	-	-	-
aGal	-	-	-
bGlu	-	-	-
	Enzymes produced (%)		8.3

Strain:	AFG20.1		
	Total activity ($nM\ min^{-1}\ mL^{-1}$)	Bound (%)	Free (%)
bG	-	-	-
Pho	10 ± 2	100	-
Lip	187 ± 32	54	46
bM	-	-	-
aA	13 ± 4	100	-
bX	-	-	-
bGal	403 ± 27	97	3
CBH	-	-	-
aG	-	-	-
ChTN	-	-	-
aGal	-	-	-
bGlu	-	-	-
	Enzymes produced (%)		33.33

Strain:	AFG22		
	Total activity ($nM\ min^{-1}\ mL^{-1}$)	Bound (%)	Free (%)
bG	43 ± 3	-	100
Pho	19 ± 2	100	-
Lip	754 ± 121	65	35
bM	-	-	-
aA	-	-	-
bX	-	-	-
bGal	13 ± 2	-	100
CBH	-	-	-
aG	-	-	-
ChTN	83 ± 3	85	15
aGal	-	-	-
bGlu	24 ± 4	-	100
	Enzymes produced (%)		50

Strain:	AFG27.1		
	Total activity ($nM\ min^{-1}\ mL^{-1}$)	Bound (%)	Free (%)
bG	-	-	-
Pho	38 ± 4	100	-
Lip	349 ± 23	38	62
bM	-	-	-
aA	-	-	-
bX	-	-	-
bGal	235 ± 14	88	12
CBH	-	-	-
aG	215 ± 4	-	100
ChTN	-	-	-
aGal	-	-	-
bGlu	12 ± 0	-	100
	Enzymes produced (%)		41.7
